# Deciphering the shape and deformation of secondary structures through local conformation analysis

**DOI:** 10.1186/1472-6807-11-9

**Published:** 2011-02-01

**Authors:** Julie Baussand, Anne-Claude Camproux

**Affiliations:** 1 Molécules Thérapeutiques in silico, UMRS-973, Université Paris-Diderot Paris-7,36, rue Hélène Brion, 75013 Paris, France

## Abstract

**Background:**

Protein deformation has been extensively analysed through global methods based on RMSD, torsion angles and Principal Components Analysis calculations. Here we use a local approach, able to distinguish among the different backbone conformations within loops, *α*-helices and *β*-strands, to address the question of secondary structures' shape variation within proteins and deformation at interface upon complexation.

**Results:**

Using a structural alphabet, we translated the 3 D structures of large sets of protein-protein complexes into sequences of structural letters. The shape of the secondary structures can be assessed by the structural letters that modeled them in the structural sequences. The distribution analysis of the structural letters in the three protein compartments (surface, core and interface) reveals that secondary structures tend to adopt preferential conformations that differ among the compartments. The local description of secondary structures highlights that curved conformations are preferred on the surface while straight ones are preferred in the core. Interfaces display a mixture of local conformations either preferred in core or surface. The analysis of the structural letters transition occurring between protein-bound and unbound conformations shows that the deformation of secondary structure is tightly linked to the compartment preference of the local conformations.

**Conclusion:**

The conformation of secondary structures can be further analysed and detailed thanks to a structural alphabet which allows a better description of protein surface, core and interface in terms of secondary structures' shape and deformation. Induced-fit modification tendencies described here should be valuable information to identify and characterize regions under strong structural constraints for functional reasons.

## Background

Our understanding of protein interaction mechanisms relies on the analysis of protein-protein complexes aiming to identify and characterize the fundamental physico-chemical and structural factors that are required for the specific recognition and functional interaction of protein partners. Considerable efforts have been made to describe protein-protein interfaces in terms of amino acids composition and evolution [1-5], and in terms of structural [6-10] and dynamical features [11-13]. The analysis of protein complexes revealed that, although specific protein-protein interfaces present distinct features compared to non-specific interfaces observed in proteins crystals [14-16], their properties can differ between the different types of complexes (i.e. homocomplexes, heterocomplexes, obligate and transient complexes) [1,10,17-20]. The analysis of secondary structures at protein-protein interface emphasized the importance of non-regular secondary structure (loops) compared to more rigid regular ones (*α*-helices and *β*-strands) preferred in the core [21]. The secondary structure percentages at interface are more correlated with those of the exterior residues which suggests that the interface is structurally closer to the protein surface than to the protein core [22]. Loops, which are more able to adjust themselves upon interaction, generally contribute to 40% of the interface [10,23]. Compared to other complexes, transient complexes present a greater involvement of loops at interface since they provide more flexibility for the protein molecules to associate and dissociate appropriately [17]. *α*-helices are also well represented at protein-protein interface, particularly in obligatory homocomplexes of which interfaces are mainly composed by helix-helix pairing [10,17]. In transient heterocomplexes, binding sites have preference for *β*-sheets and long non-regular structures but not for *α*-helices [8]. The strong preference for *β*-sheets is probably due to their high ability to form densely packed structures when placed one against the other, thus having a higher potential for intermolecular bond formation. In addition, secondary structures appear to be under constraints to form interface scaffolds favorable to protein-protein interaction [24].

Besides the static structural description of protein-protein interfaces, conformational and dynamical changes upon complexation have been analysed since they have important implication for the development of docking algorithms [25]. Both the 'induced-fit' [26] and the 'pre-existing equilibrium' [27] models for protein binding mechanism underline structural differences between the bound and unbound states of proteins. In the former model the differences are due to conformational changes induced by the binding of the ligand, while in the latter the differences are more related to dynamical changes where the bound state corresponds to conformations that pre-exist in the unbound conformations ensemble. Comparisons between bound and unbound structures have been mainly performed through RMSD, torsion angles [11,28], RMSF and Principal Components Analysis calculations [12]. Evidence for both models have been found possibly playing a joint role in molecular recognition [29,30]. Structural differences between the bound and the unbound states of a protein can be either large (monoclonal IgE antibody, RMSD ~ 7Å) or small (less than 1Å). Conformational changes are not restricted to the interface and affect around 20% of the residues in allosteric proteins [11,28]. Interface residues generally undergo larger motions than the rest of the protein in the case of enzymes [31]. In the case of ubiquitin, local structural variations in the region surrounding the binding site have been found to play an important functional role allowing the protein to adapt to its several structurally diverse partners despite a low RMSD in the ensemble of the recognition dynamics [30,32]. The importance of the local structural variation observed in the binding process of ubiquitin highlights the need for efficient local approaches to understand the mechanism of protein-protein interaction. In terms of dynamics, mobility of residues at interface is not homogeneous, core and surface interface residues are respectively less and more mobile than the rest of the surface [12,13]. In terms of secondary structures elements, loops are more likely to experience motions than *α*-helices and *β*-strands [28]. Although the secondary structure composition at protein-protein interface is similar in bound and unbound conformations [8], changes in secondary structures from disorder-to-order and order-to-order occur, possibly playing important functional roles [33].

An innovative way to analyse and characterize induced-fit conformational changes has been proposed which consists of translating the 3 D protein structures into 1 D structural sequences using a structural alphabet [34]. What is the advantage of using a structural alphabet to analyse secondary structures shape and their induced-fit deformation? Helical secondary structures can be curved, kinked or straight [35]. Strand geometry depends on sheet parallelism and pleat which results in variable conformation of the *β*-strands. Loops are weakly constrained structures and therefore difficult to characterize and compare. The HMM-SA structural alphabet [36] describes the local shape of proteins and the logic of their assembly in 27 structural letters. It provides a detailed description of the protein backbone and allows the identification of conformational variations within the different secondary structure types. We call conformational variations differences in the backbone conformation (modeled by different structural letters) leading to variation in the shape of the secondary structures. Four structural letters are associated with variation in the backbone of *α*-helices, five to variation in the backbone of *β*-strands. The 18 remaining structural letters described local conformations forming loops. Thus the structural alphabet provides a way to distinguish among the different conformational states of each type of secondary structure, and also to characterize these states being then comparable. The study presented in [34], in which HMM-SA was used to analyse the differences in structural letter composition at interface of bound and unbound proteins, was the first qualitative description of induced-fit structural changes. It revealed that some specific local conformations in coils are more likely to be deformed at interface upon complexation than other, and that the severity of the structural changes may also vary.

Here we investigate the structural differences between the local conformations that can explain this variable behavior in respect of deformation upon complexation. While the previous study mainly focused on the deformation at interface of local conformations associated with loops, here we analyse each of the three types of secondary structure in the whole proteins. We first verify that the structural alphabet is able to fit previously reported description of protein interface, surface and core in terms of the secondary structure for the four different types of complexes. A more detailed analysis reveals a non-uniform distribution of the structural letters within proteins with clear preference of particular structural letters for either surface or core, and to a lesser extent for interface and non-interface regions. We show that structural letters with similar distribution preference shared common structural and solvent exposure features. In other words, it means that different backbone conformations tend to be adopted by the secondary structures depending on their location in proteins at interface, on surface or in core. We revisit the analysis of the structural deformation of local conformations upon interaction proposed in [34] by comparing a dataset of bound and unbound proteins and show how the deformation of local conformations is related to their preferred location in proteins. Deformation tendencies for local conformations are defined and different example cases of deformation are presented.

## Results and Discussion

### HMM-SA encoding and secondary structures

**Figure 1:**
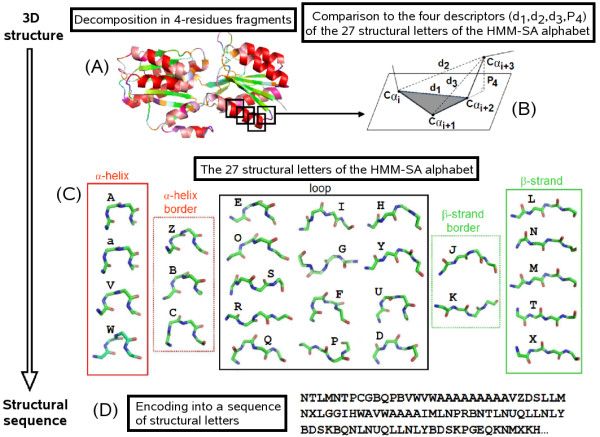
**From the 3 D structure to the 1 D structural sequence**. A protein structure is decomposed into overlapping four-residue fragments (A). Each fragment is described by a vector of four descriptors *d*_1_, *d*_2_, *d*_3 _and *P*_4 _(B) allowing their comparison to the 27 structural letters of the HMM-SA alphabet presented in (C) where *α*-letters (red frame), *β*-letters (green frame), borders-letters associated with helix-borders (red dotted frame) and to strand-borders (green dotted frame) and loop-letters (black frame) are indicated. The encoding of a 3 D structure into the structural sequence (D) takes into account both the similarity of the protein fragments with the 27 structural letters and the preferred succession of the structural letters.

HMM-SA is a library of 27 structural prototypes (structural letters) of four *α*-carbons named [A-Z,a] [36]. HMM-SA allows the 3 D structure of a protein backbone to be decomposed in four-residue fragments, each of them being described by four descriptors relying on inter-C*α *distances. More precisely, it corresponds to the distances between the *α*-carbons of residues 1 and 3 (*d*_1_), of residues 1 and 4 (*d*_2_) of residues 2 and 4 (*d*_3_) and to the oriented projection of the last *α*-carbon to the plane formed by the three first ones (*P*_4_). The resulting descriptors are the input of an hidden Markov model able to encode any low energy structure of a protein into its corresponding structural letters sequence (Figure 1 and Additional file 1). The encoding takes into account both the similarity of the protein fragments with the 27 structural letters and the preferred transitions between the structural letters [36,37]. Secondary structures of protein are assigned related to their HMM-SA encoding, as in [38]. The four structural letters [a,A,V,W] describe the different local conformations associated with *α*-helices (denoted *α*-letters), the five structural letters [L,M,N,T,X] are associated with *β*-strands (denoted *β*-letters), the 13 letters [D,E,F,G,H,I,O,P,Q,R,S,U,Y] are associated with loops (denoted loop-letters) and the five letters [Z,B,C] and [J,K] are associated with *α*-helix and *β*-strand borders (denoted border-letters). Although classical secondary structure assignment methods attribute residues to either regular or non-regular secondary structures, secondary structures borders are transitional conformations between the two and can be characterized by the structural alphabet. They are classified as loops initially but are analysed separately in the following.

### Distribution of secondary structures within protein compartments

Proteins of large datasets of protein-protein complexes were decomposed into three compartments: core, interface and surface. The residue distribution among the three protein compartments fits with the one reported in [39] (Additional file 2). The mean number of interface residues per complex is smaller in heterodimers (30.6 ± 16.4) and transient complexes (21.4 ± 8.2) than in homodimers (43.3 ± 23.5) and obligate complexes (44.9 ± 21.9) respectively, in agreement with [17,40,41]. Secondary structure distribution is evaluated according to the secondary structure type of the structural letters within the three compartments (Table 1). The large majority of structural letters on surface and at interface corresponds to non-regular conformations (border- and loop-letters), while in core they are mainly associated with regular ones (*α- *and *β*-letters). The great number of loop- and *α*-letters at interface compared to *β*-letters in homodimers and obligates complexes, as well as the greater proportion at interface of *β*-letters compared to *α*-letters in heterodimers and transient complexes, is consistent with [8,10,22]. Secondary structure distributions at interface, surface and core compartments are maintained in proteins between bound and unbound states as previously reported in [8]. We show here that the local approach is as reliable as the global one since similar observations are made on secondary structure distribution at interface, surface and core for the different types of complexes. In the following, protein-protein complexes are further explored with the local approach by distinguishing among the different structural letters of the same secondary structural type.

**Table 1:** Secondary structures distribution at protein interface, surface and core

	Interface	Surface	Core	All
*Complete dataset*				
*α*	25.7	26.8	32.8	28.6
*β*	18.4	15.9	32.6	21.7
loop	36.3	37.4	23.0	32.6
border	19.6	16.8	13.6	17.1

*Homodimers/Heterodimers*				
*α*	24.3/19.5	27.1/21.2	32.3/28.5	28.2/22.6
*β*	18.8/25.2	15.1/20.4	32.1/38.7	21.1/26.0
loop	37.3/36.9	37.6/38.7	23.8/22.3	33.2/34.3
border	19.6/18.4	20.2/19.6	11.1/10.5	23.6/17.1

*Obligate/Transient*				
*α*	23.5/17.6	26.0/17.4	31.7/24.0	27.5/19.3
*β*	18.6/22.7	15.0/23.3	29.7/38.9	20.4/27.8
loop	37.5/40.8	38.2/39.9	25.4/26.7	33.8/36.2
border	20.4/18.9	20.8/19.3	13.2/10.4	18.2/16.7

*Bound/Unbound*				
*α*	15.0/15.0	17.6/17.8	23.7/24.0	19.1/19.4
*β*	18.2/17.7	21.9/22.0	39.7/40.1	26.9/27.1
loop	46.4/46.8	40.1/40.0	25.1/24.8	36.2/36.2
border	20.2/20.3	20.3/20.0	11.4/11.0	17.6/17.3

Percentage of secondary structures in the three protein compartments Interface, Surface, Core and their global proprotion in proteins (All) are given for the five datasets. Regular secondary structures are evaluated by *α- *and *β*-letters, non-regular ones by loop- and border-letters.

### Distribution of local conformations within protein compartments

**Figure 2:**
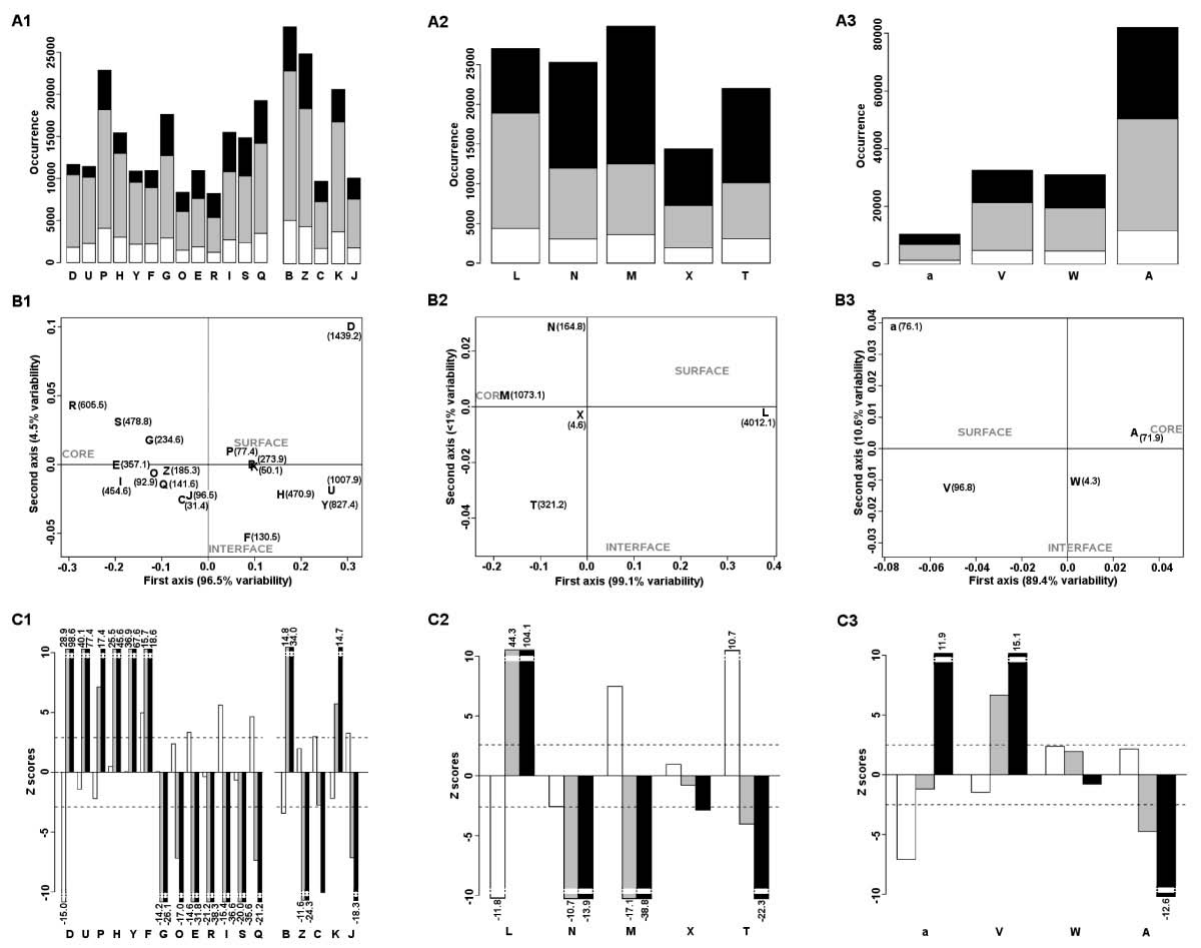
**Statistical analysis of structural letters distribution in the complete dataset**. Analysis of border- and loop-letters (column 1), *β*-letters (column 2) and *α*-letters (column 3) is given. A) Occurrence of structural letters in the three protein compartments: interface (white), surface (grey) and core (black). B) Multiple Correspondence Analysis performed on the occurrence of the structural letters in the three protein compartments. KLd quantities are indicated in parenthesis, statistical significance is reached for a value > 5.99. The first axis is associated with variabilities from 89.4% up to 99% separating surface and core, the second axis are associated with variabilities from 1% up to 10.6% separating interface and non-interface regions. C) Z-score values assessing the preference of structural letters for the interface compared to the surface (*Z_inter face/sur face_*: white), for the interface compared to the core (*Z_inter face/core_*: grey) and for the surface compared to the core (*Z_sur face/core_*: black). Statistical significance thresholds after Bonferoni correction are indicated by dashed lines. It corresponds to |2.5| for *α*-letters, |2.6| for *β*-letters and |2.9| for loop-letters. Z-score values> 2.5 correspond to p-values < 6.10^-3^, Z-score values > 6 correspond to p-values< 10^-11^.

Compartment preference of secondary structures is further deciphered by analysing the distribution of each structural letter among the three compartments. Although *β*-, loop- and border-letters are similarly represented in proteins, *α*-letters present important representativeness differences (Figure 2A). Moreover the distribution of letters associated with the same secondary structure type differs in the three protein compartments (Figure 2A) and is precisely analysed in a qualitative (Multiple Correspondence Analysis MCA) and statistical (Kullback-Leibler divergence KLd and Z-score measures) manner (Figure 2B). The MCA performed on loop-/border-, *α- *and *β*-letters shows that the most informative axis distinguishes between core and surface (from 89.4% to 99% of variability associated with the first axis, Figure 2B). Differences between interface and non-interface are less discriminative on the MCA plots (1% to 10.6% variability associated with the second axis, Figure 2B) but Z-score values assess significant preference for some letters (Figure 2C). A detailed analysis for each set of structural letters corresponding to the different secondary structures is presented below.

#### Distribution of loop-letters and border-letters

The first axis of the MCA plot separates loop-letters into two groups of letters (Figure 2B1, C1): [G,R,S,O,E,I,Q] and [P,H,Y,U,D,F] preferentially distributed in core and on surface respectively. In addition, some letters show a preference for interface or non-interface regions (Figure 2C1). In the first group, [E,I,Q,O] present preference for interface (positive *Z_inter face/surface_*) with significant Z-score values for [E,I,Q]. In the second group, [D] is under-represented at interface (highly negative *Z_inter face/surface_*) whereas [F] shows preference for interface. The KLd values associated with border-letters are all significant: [B,K] are the most preferred on surface and the least in core while [Z,C,J] display the opposite behavior.

#### Distribution of β-letters

Non-uniform distribution among the three protein compartments is also observed for *β*-letters (Figure 2B2,C2). Letter [L] obtains the most significant KLd value among the 27 structural letters and displays a clear preference for surface. Significant KLd values are obtained for *β*-letters [M,N,T] which are preferentially distributed in core as illustrated by the MCA plot. Letters [T,N] are clearly distinguished by the second axis of the MCA plot: letter [T] is preferred at interface compared to surface while [N] is under-represented at interface compared to both surface and core indicating its preference for non-interface regions. Letter [X] has no significant preference.

#### Distribution of α-letters

Letters [A,a,V] exhibit different distribution in the three compartments (Figure 2B3, C3) while letter [W] has no clear preference. Letter [A] is preferred in core while [a,V] are preferred on surface. More precisely, Z-scores show the preference of [a] for non-interface region being preferred in both core and surface compared to interface (Figure 2C3). Notice that the KLd and Z-score values obtained for *α*-letters are lower than the ones obtained for loop- and *β*-letters indicating that *α*-letters display weaker distribution differences than the other structural letters.

### Compartment preferences in the different types of protein-protein complexes

The distribution analysis of the structural letters in the three protein compartments of the complete dataset unveils compartment preferences among local conformations belonging to the same secondary structure type. The local approach analysis reveals a tendency for secondary structures to adopt different local shapes according to their location in proteins at interface, surface or core. The analysis of homodimers, heterodimers, obligate and transient complexes separately shows a similar distribution preferences for local conformations among the different types of complexes (Additional files 3, 4, 5 top and center). In particular, the distribution preference of letters for surface, core and non-interface is very strong and stable while the preference of letters for interface is more likely to vary between the different complexes. However, for transient complexes, the preference of local conformations for interface and non-interface is maintained in both bound and unbound states suggesting a structural predisposition of binding sites for interaction (Additional files 3, 4, 5 bottom).

In order to quantify the extent of the preferential distribution of secondary structures in proteins, the difference between the observed occurrence of a letter in a compartment and its expected occurrence (calculated with the proportion of the secondary structure type in the compartment) over the observed occurrence in a compartment of a letter (Table 2) is computed. The proportion of structural letters affected by the preferential distribution is evaluated for the different types of protein-protein complexes and is shown to be consistent varying between 12-17% for loop-letters, 4-9% for border-letters, 13-23% for *β*-letters and 3-7% for *α*-letters (Table 2).

**Table 2:** Percentage of secondary structures affected by the preferential distribution

Dataset	Interface	Surface	Core	All
*α-letters*				
Complete	2%	3%	4%	3%
Homodimers/Heterodimers	5%/3%	4%/5%	6%/6%	5%/5%
Obligate/Transient	9%/4%	4%/4%	4%/5%	4%/4%
Ubound/Bound	9%/9%	5%/2%	9%/6%	7%/4%

*β-letters*				
Complete	10%	35%	17%	23%
Homodimers/Heterodimers	12%/11%	17%/12%	17%/17%	16%/14%
Obligate/Transient	10%/18%	20%/10%	18%/15%	18%/13%
Ubound/Bound	17%/17%	15%/14%	17%/18%	16%/16%

*loop-letters*				
Complete	7%	10%	29%	14%
Homodimers/Heterodimers	8%/6%	9%/8%	29%/36%	13%/12%
Obligate/Transient	7%/11%	9%/8%	23%/30%	12%/13%
Ubound/Bound	12%/12%	11%/11%	38%/38%	17%/16%

*border-letters*				
Complete	2%	5%	16%	7%
Homodimers/Heterodimers	3%/4%	6%/4%	17%/8%	8%/4%
Obligate/Transient	4%/10%	7%/3%	17%/6%	9%/5%
Ubound/Bound	12%/7%	5%/5%	13%/14%	7%/7%

The percentage of secondary structure affected by the preferential distribution is evaluated in the Interface, Surface and Core compartments using the difference between the observed number of the structural letters in a compartment and its expected number (given the repartition of the secondary structures type in the three compartments). The total effect (All) is evaluated according to the sum of the difference in the three compartments. The evaluation is performed on the seven datasets.

The local approach reveals that some local conformations are more affected by the preferential distribution than others. For instance structural letters [L] and [M], which have been shown to be preferred on surface and in core respectively, correspond to 57% of the *β*-letters affected by the preferential distribution in the complete dataset (Additional file 6).

In the following, *α*-letters [a,V], *β*-letter [L], loop-letters [P,H,Y,D,U,F] and border-letters [B,K], which are local conformations preferentially distributed on surface, are grouped together as *surface-letters*. Strong preference for core is observed for *α*-letters [A], *β*-letters [T,M,N], loop-letters [G,R,O,I,S,E,Q] and border-letters [Z,C,J]. They are therefore grouped together as *core-letters*. Although the representation at interface of some letters may vary among the different types of complexes, the tendency for letters [F] and [a,N,D] to be preferred in interface and non-interface regions respectively is very stable. Letters [a,N,D] are then further characterized as *non-interface-letters *and letter [F] as *interface-letter*. The structural characteristics of these groups of local conformations are analysed.

### Compartment preference and amino acids composition of local conformations

The amino acids composition of local conformations is evaluated at interface, surface and core in the complete dataset. For each structural letter, tryptophan and tyrosin are in greater or similar proportion at interface than in core while all other hydrophobic residues present a greater proportion in core. Arginine and histidine present their highest proportion at interface compared to both surface and core. These residues have been previously found to be enriched at protein interface [1,8,42]. The proportion of proline and glycine, two residues known to be key structural residues, is observed to greatly vary between some structural letters, however these differences do not distinguish between *surface*- and *core-letters *structural letters (Additional files 7 and 8). *Interface-letter *[F] presents a high proportion of both residues (14% of proline and 22% of glycine at interface). *Non-interface-letters *[a,N] present low proportion of proline (from <7%) while [D] appears to be particularity enriched in glycine (55%) in agreement with [37]. Other structural letters with different compartment preference [J,R,U] are enriched in glycine. Then the amino acid composition of the structural letters, analysed in the different compartments, is unlikely to explain the compartment preference of the local conformations and confirms that amino acids and local conformations give complementary and not redundant information.

### Compartment preference and structural description of local conformations

**Figure 3:**
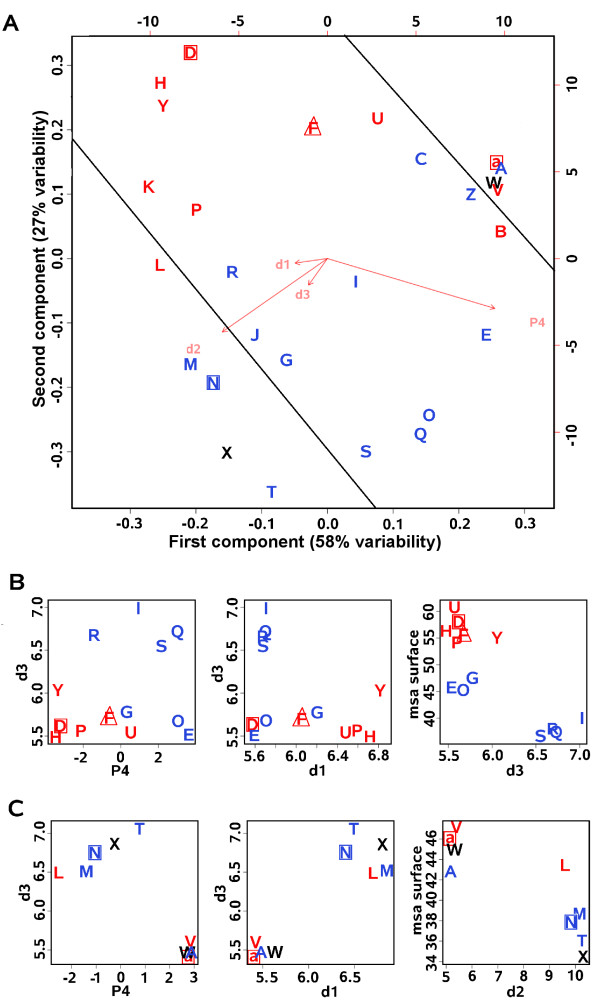
**Structural characteristics of the structural letters**. Structural descriptors analysis of *surface-letters *(red), *core-letters *(blue), *non-interface-letters *(square), *interface-letter *(triangle) and non characterized ones (black). A) Principal Component Analysis performed on the structural descriptors *d*_1_, *d*_2_, *d*_3 _and *P*_4 _of the 27 structural letters. The first component is associated with 58% of variability, the second one to 29% of variability and the third and fourth axis to 7% and 6% respectively. Plain lines separate letters according to their secondary structural types. B) Correlation plot *d*_3_/*P*_4_, *d*_3_/*d*_1 _and msa/*d*_3 _for loop-letters. Msa (Mean solvent accessibility) are computed for the surface compartment but similar observations are made for the interface compartment. C) Correlation plots *d*_3_/*P*_4_, *d*_3_/*d*_1 _and msa/*d*_2 _for *α- *and *β*-letters. Msa stands for the mean relative solvent accessibilities, it is calculated for each letter in the surface compartment of the complete dataset.

A Principal Component Analysis (PCA) is performed on the four structural descriptors characterizing the 27 structural letters of the structural alphabet (Figure 3A and Table S1). The first component (58% of variability) is strongly associated with descriptor *d*_2 _and inversely to *P*_4 _(characterizing respectively the total length and volume/orientation of the local conformation, see Figure 1) with few importance to *d*_1 _and *d*_3 _(length between the three first and last *α*-carbons, see Figure 1). It differentiates letters according to their secondary structural types: *β*-letters are the most extended (long *d*_2_), *α*-letters are the least ones with large volume (short *d*_2_, large *P*_4_) and loop-letters present variable conformations (intermediate *d*_2 _and *P*_4_) with border-letters being the closest to the *α- *and *β*-letters. Unsurprisingly, the secondary structure type of the letters is the most important structural factor differentiating the conformation of the different structural letters. The second component of the PCA (27% of variability) is positively associated with the descriptors *d*_2 _and *P*_4 _and positively to descriptor *d*_3 _in a minor way (Figure 3A). It appears From the PCA plot, it appears that the structural letters can be discriminatedz according to their preference for surface or core compartments (Figure 3A) suggesting that specific structural features, captured by the structural descriptors, are related to solvent exposure. A detailed analysis for non-regular and regular structural letters is presented below.

#### Characteristics of loop-letters

By focusing on the values of descriptors *P*_4_/*d*_3 _and *d*_1_/*d*_3 _for loop-letters (Figure 3B), we observe that *surface-letters *associated with loops correspond to local conformations with short *d*_3 _and a tendency for low or negative *P*_4_. *Non-interface-letter *[D] and *interface-letter *[F] differ from the other *surface-letters *with the shortest *d*_1_. *Core-letters *display short *d*_1 _with positive *P*_4 _but can be separated in two groups: [I,R,S,Q] display long *d*_3 _while [G,E,O] display short *d*_3 _comparable to *surface-letters*. These structural differences between the loop local conformations agree with their solvent accessibility (Figure 3B right). All *surface-letters *as well as *core-letters *[I,R,S,Q] are respectively the most and least accessible to solvent while *core-letters *[G,E,O] present intermediate solvent accessibility. It suggests that local conformations with short *d*_1 _and long *d*_3 _are related to unfavored solvent exposure and then preferentially distributed in core, while local conformations with long *d*_1 _and short *d*_3 _are more exposed to solvent with variation according to the extent of the curvature (variation in *d*_3 _values) and its orientation. A negative *P*_4 _appears to indicate an orientation towards the protein exterior and is associated with *surface-letters *while positive *P*_4 _indicates an orientation towards the protein interior and is associated with *core-letters*. Notice that *border-letters *present intermediate descriptor values since they can be associated with either regular or non-regular conformations, and so are not considered here.

#### Characteristics of β- and α-letters

Similarly for *β*-letters (Figure 3C), *surface-letter *[L] is significant of a curvature in *β*-strands (the shortest *d*_3 _and highly negative *P*_4_) and presents the highest solvent exposure on surface among all *β*-letters, while *core-letters *[T,X,M,N] are the least exposed. In particular, [T,M] correspond to straight *β*-strand conformations (with the large *d*_2_). Distinction between *α*-letters in terms of structural descriptors is not clear (Figure 3A,C), which is coherent with the fact that they also display the least differences in terms of distribution between the three protein compartments (Figure 2). However, their subtle differences in terms of structural descriptors are in fact reflecting different helix geometries: *surface-letters *[V,a] are associated with distortions leading to kinked and curved helices respectively while [A] forms straight helices [36]. *Non-interface-letters *[a,N] also display common structural specificities corresponding to the local conformations with the shortest *d*_1 _in respect with the other letters of the same secondary structure type. The structural specificities of letters associated with either regular or non-regular secondary structures but sharing the same compartment preference are unveiled: curved conformations appear to be preferred in surface and straight ones in core. Such variations in the backbone of secondary structures is associated with solvent exposure differences. Local conformations avoided at interface correspond to conformations with the shortest distance C_*α*1_-C_*α*3_. These results reveal new structural features, regarding the preferential shape of regular and non regular secondary structures in proteins compartments, which have not been appreciated before.

### Revisiting the deformation of local conformations

The deformation of local conformations upon complexation previously studied in [34] is revisited and results are further interpreted in the light of the compartment preference and structural characteristics of the local conformations. We use a protein-protein interface definition based on solvent accessibility variation (versus contact points with voronoi tessellation) and consider all structural letter transitions (versus only severe deformations with local RMSD greater than 0.2Å) within and between the different secondary structure types.

#### Deformation of local conformations

**Figure 4:**
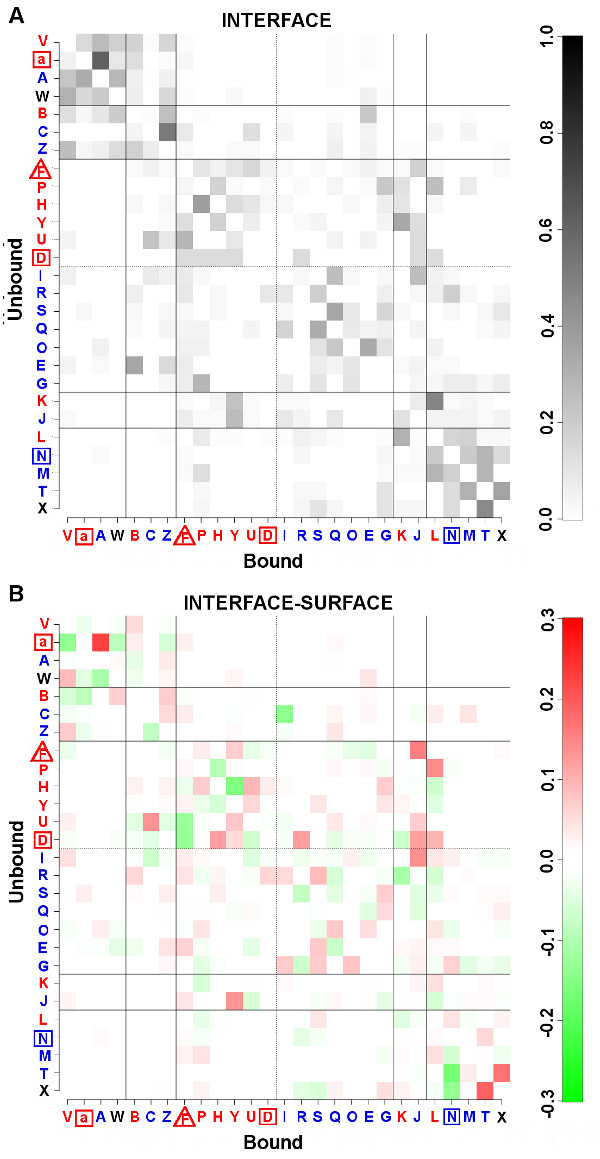
**Deformation matrices**. A) Matrix of deformation proportion *P *(*sl*_1_, *sl*_2_) at interface (see method) where *sl*_1 _is the letter in the unbound state (y-axis) and *sl*_2 _the corresponding letter in the bound state (x-axis). B) Matrix of deformation differences between the interface and the surface Δ*P*(*sl*_1_, *sl*_2_) (see method) where *sl*_1 _is the letter in the unbound state (y-axis) and *sl*_2 _the corresponding letter in the bound state (x-axis). For the two matrices, the black lines separate structural letters according to their secondary structure type. The structural letters are differentiated according to their compartment preferences (blue for core, red for surface, triangle for interface and square for non-interface). Dotted lines separated surface loop-letters from core ones.

The deformation of secondary structures is analysed by comparing the structural letter transitions from the proteins unbound to bound state. The local conformations are mainly unchanged in the three compartments, the majority of deformation occurred at interface (38% of the structural letters are changed between the bound and unbound states) compared to surface (34%) and core (30%) in agreement with [34]. At interface, 66% of *α*-letters, 39% of *β*-letters and 27% of loop-letters are changed, among which 73%, 65% and 60% of *α*-, *β- *and loop-letters respectively are changed for letters of the same secondary structural type (Figure 4A). But interestingly, on the other hand, the proportion of changed border-letters corresponds to 75% and are changed towards loop-letters (32%), *α*-letters (28%) and *β*-letters (15%). It highlights that, although secondary structures are very stable upon complexation (in agreement with [8]), their borders are more likely to be deformed or adjusted upon interaction. Similar observations are made for the surface and core compartments, however the proportion of *α- *and *β*-letters that are changed for letters of the same structural type is even higher with 87% and 81% respectively (Additional file 9). Analysing the substitutions of each structural letter at interface gives a more detailed picture of secondary structure deformations upon complexation (Figure 4B). For *α*-helices, curved *non-interface-letter *[a] (the most changed *α*-letter: 80%) displays a clear preference to be deformed towards straight *core-letter *[A] upon interaction (the least changed *α*-letter: 60%) while the inversed substitution is more likely to be due to protein flexibility being as observed at interface as in surface. Similarly for *β*-strands, *non-interface-letter *[N] (the most changed *β*-letter: 48%) is preferentially deformed towards the straightest *core-letters *[T,M] (the least changed *β*-letters: 29% and 34% respectively). Curved *surface-letter *[L] (deformed in 42% of cases) appears to be deformed towards [N]. For loop-letters, *non-interface-letter *[D] is the least changed letter (11%) and *core-letter *[R] the most one (45%). The fact that the least changed loop-letter [D] corresponds to a conformation avoided at interface suggests a non-flexible conformation interfering with efficient recognition or interaction with the other protein. 27% of the *interface-letter *[F] are deformed. No clear preferential deformation appears between specific loop-letters but they appear to be deformed towards letters with the same compartment preference: 70% of *surface-letters *[D,U,P,H,Y,F] are changed towards *surface-letters *and 75% for *core-letters *[G,R,O,I,E,S,Q] are changed towards *core-letters*. Although the deformation tendencies at interface of local conformations associated with regular secondary structures (from curved to straight conformations) agree with their compartment preference (*non-interface-letter *and *surface-letter *are deformed towards *core-letters *when interface residues become buried upon complexation), the expected deformation of loops from *surface-letters *to *core-letters *is not observed. Instead deformation appears to be barely affected by solvent accessibility variation induced by the complexation with transitions between local conformations of the same compartment preference/structural characteristics. The relation between loop deformation and exposure to protein exterior is further analysed.

#### Deformation of loops and exposure to protein partner

Relative solvent accessibilies are computed for deformed local loop conformations in the interface compartment in both unbound and disjoint bound conformations, and the difference *D *between the two accessibilities is calculated. A negative difference indicates a deformation towards a local conformation with higher exposure to the exterior (i.e. towards the partner) while a positive one indicate a tendency for lower exposure. The average difference $\bar{D}$ calculated on *surface-letters *deformed on *surface-letters *(${\bar{D}}_{s/s}$ = -8.2 ± 22.6%, median = -5.0) and on *core-letters *deformed on *core-letters *(${\bar{D}}_{c/c}$ = -1.5 ± 18.1%, median = -2.6) are all negative indicating that complexation globally increases residue exposure to the protein exterior. However, deformation of *surface-letters *towards *surface-letters *tend to be associated with higher exposure than deformation towards *core-letters *(${\bar{D}}_{s/c}$ = -4.5 ± 24.7%, median = 1.3). Coherently, deformation of *core-letters *towards *core-letters *tend to be associated with lower exposure than deformation towards *surface-letters *(${\bar{D}}_{c/s}$ = -11.4 ± 21.8%,median = -7.7).

Put all together it suggests that, since the deformation of loops upon complexation barely modify their exposure to protein exterior (transitions mainly between letters sharing same compartement preference and structural characteristics), most of local loop conformations are in an optimized conformation for interaction in the unbound state. More drastic deformations of local conformations occur (transitions between letters of different compartment preference and different structural characteristics) which tend to modify the exposure of the residues towards the protein partner. Transitions from a *core-letter *to a *surface-letter *at interface would favor residue interaction between the two partners (increase exterior exposure) while the reverse transitions tend to unfavor it (decrease exterior exposure).

#### Deformation tendencies

Local conformations are not subject to the same rate of deformation and follow some specific deformation tendencies: i) transitions from one secondary structure to another are avoided but deformation within each secondary structure type occur with preferences between pairs or groups of letters ([a]→ [A] for helices, [N]→ [T,M] for strand, [P,H,Y,D,U,F]→ [P,H,Y,D,U,F] and [G,R,O,I,S,E,Q]→ [G,R,O,I,S,E,Q] for loops), ii) deformation preference between local conformations are not commutative, iii) flanking regions are the most frequently deformed local conformations. These observations are in agreement with [34]. The analysis of the distribution of local conformations in proteins highlights new features, and their deformations are consistent with their compartment preferences. Regarding regular secondary structures, iv) the most deformed local conformations [a,N] correspond to curved conformations which tend to be avoided at interface (in both bound and unbound states), v) the least deformed ones [A,T,M] correspond to straight conformations preferentially distributed in core and vi) the most deformed local conformations tend to be preferentially deformed towards the least deformed ones. Regarding loops, vii) two groups of local conformations emerge where deformation preferentially occur between local conformations of the same group, viii) these two groups present different compartment preference, one being preferred in core and the other on surface, ix) deformation from one group to the other is associated with higher variation of protein exterior exposure than deformation between local conformations of the same group.

Notice that the correlated straightening-out of regular secondary structures on each side of the interface of the complexes has been evaluated through the occurrence difference of regular straight letters [A,T,M] between the unbound and bound states of each subunit of each complex. However, the low number of observations per complex does not allow any firm conclusions to be drawn.

### Illustration of deformation captured by the structural alphabet

Example cases of protein-protein interaction are selected from the bound/unbound dataset to illustate the information that can be derived from the deformation tendencies described above. The two first examples illustrate induced-fit modifications that follow the deformation tendencies, the last four illustrate their violation.

#### From curved to straight regular secondary structures

**Figure 5:**
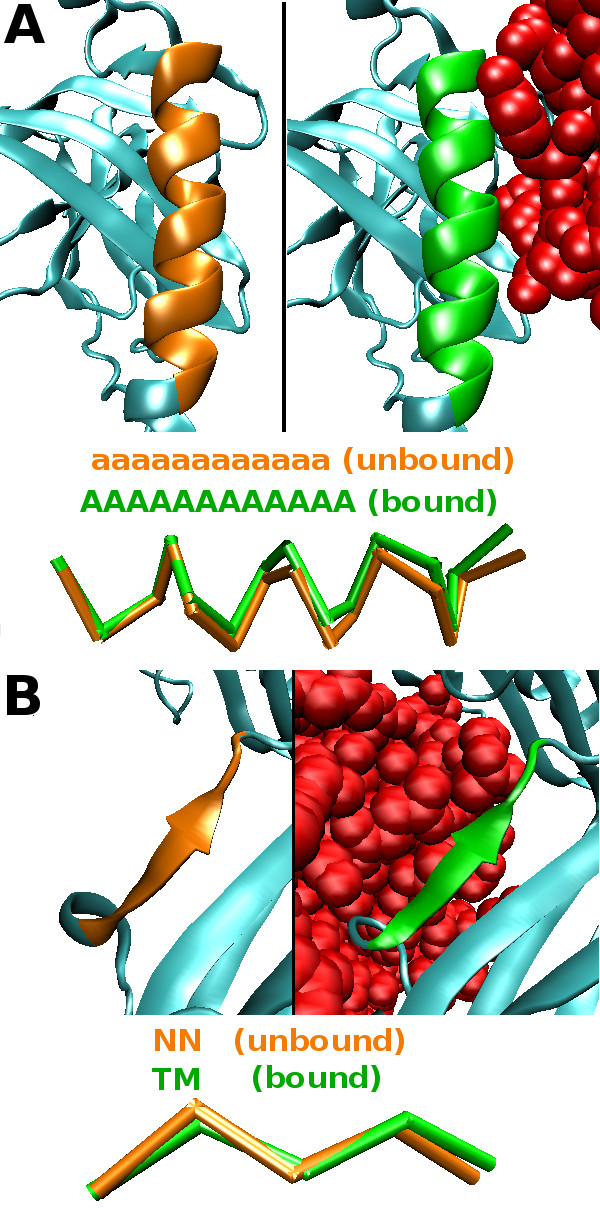
**Deformation of regular secondary structures fitting the deformation tendencies**. A) Example of a curved *α*-helix encoded by a run of [a] in the structural sequence (unbound, orange) towards a straight *α*-helix encoded by a run of [A] upon interaction (bound, green). B) Example of a curved *β*-strand encoded by a run of [N] in the structural sequence (unbound, orange) towards a straight *β*-strand encoded by "TM" upon interaction (bound, green).

The fifteen-residue helix of the human melanoma antigen complexes interacting with an enterotoxin ([PDB:1KLU], chain A:58-72) displays a C*_α _*RMSD of 0.26Å between its bound and unbound conformations (calculated with MATRAS [43]). It illustrates the deformation of a curved *α*-helix (run of [a]) towards a straight one (run of [A]) (Figure 5A). The five-residue *β*-strand of the CD8*α*(*α*) in complex with the human Major Histocompatibility Complex molecule HLA-A2 ([PDB:1AKJ] chain D:228-232) corresponds to a curved *β*-strand (run of two [N]) in the unbound state that is deformed into a straight one ("TM") in the bound state (Figure 5B). This deformation is associated with a backbone variation of 0.66Å RMSD. In these two examples, it is likely that the interaction of the protein chains caused a pressure at the interface flattening the surface of the secondary structures. Such a mechanism would explain the deformation tendencies defined above for regular secondary structures.

All the following example cases illustrate the violation of the deformation tendencies. In these examples, it appears that the observed deformations are associated with structural constraints directly related to the function of the proteins.

#### From straight to curved helices

**Figure 6:**
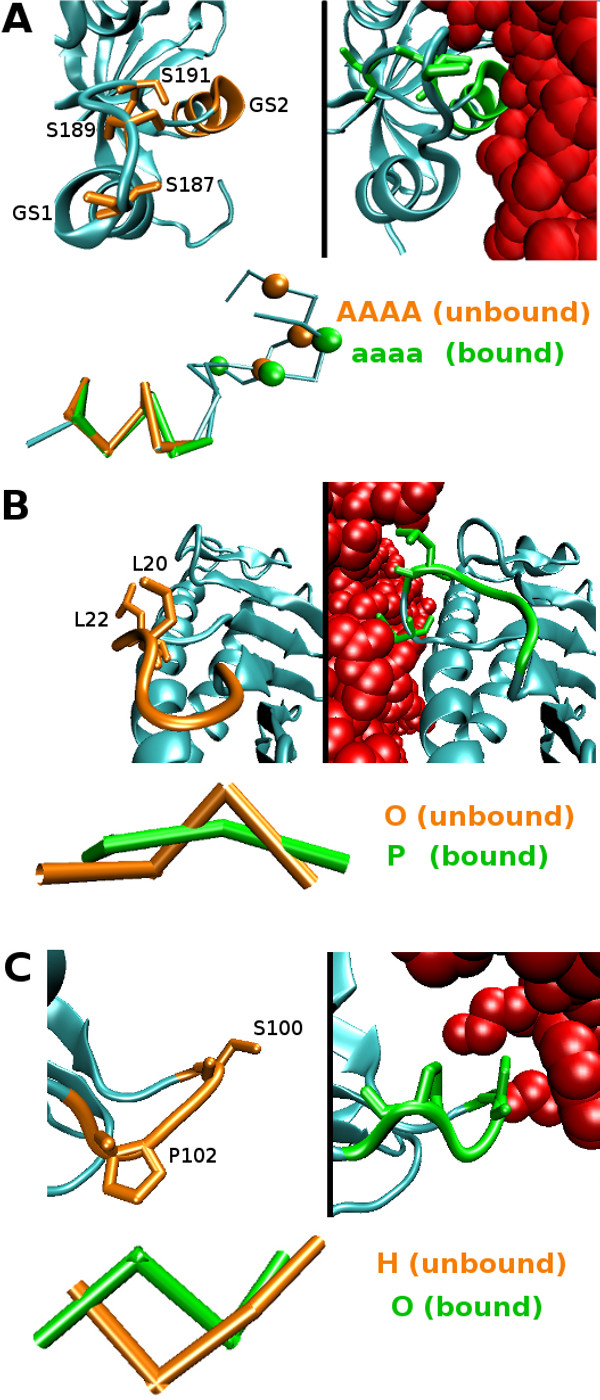
**Deformation of secondary structures not fitting the deformation tendencies**. A) The inhibitory conformation of the TGF*β *receptor in interaction with FKBP12. The structural deformation associated with the three serines (orange balls in the unbound state, green balls in the bound state) of the phosphorylation site and to the *α*GS2 from a straight *α*-helix encoded by a run of [A] in the structural sequence (orange) to a curved *α*-helix encoded by a run of [a] upon interaction (green) are represented. B) Deformation of the *α*1 domain loop of HFE upon complexation with TfR from an unbound curved (orange) to a bound extended (green) conformation enabling a higher exposure of L20 and L22 (licorice representation) to the protein exterior and the interaction of L22 with TfR (red). C) Deformation of a surface loop on the transthyretin surface upon interaction with a retinol-binding protein (red) from an unbound straight conformation (orange) to a bound curved one (green), where S100 (licorice representation) is pushed towards the protein interior upon complexation.

The first example regards the deformation of the seven-residue *α*GS2 helix of the TGF*β *receptor type I (T*β*R-I, 1B6C B:195-201) upon interaction with FKBP12, an inhibitor of the TGF*β *pathway ([PDB:1B6C] chains A, B). The phosphorylation site of the T*β*R-I is located in the GS loop surrounded by the two helices *α*GS1 and *α*GS2. When FKBP12 interacts with the *α*GS2, the helix nestles into the T*β*R-I structure and the GS loop formed an inhibitory wedge that inserts into a space in the protein core [44,45]. *α*GS2 presents a C*_α _*RMSD of 0.56Å between the unbound and bound states that the local approach reveals to correspond to the deformation of a straight conformation encoded by a run of [A] towards a curved one encoded by a run of [a] (Figure 6A). This deformation violates the deformation tendencies of *α*-helices and reveals structural constraints imposed on the *α*GS2 helix to allow the GS region to adopt an inhibitory conformation induced by the interaction with FKBP12.

#### Loop deformations

The two following examples illustrate the deformation of loops associated with transitions between *surface*-and *core-letters*, which are in violation with the deformation tendencies. Residues 18-21 ([PDB:1DE4] chain A) belonging to the *α*1 domain loop of the hemocromatosis protein (HFE) is deformed upon interaction with the transferin receptor (TfR) from a curved conformation (modeled by *core-letter *[O]) to a straight conformation (modeled by *surface-letter *[P]) (Figure 6B). This extended conformation of the loop allows the exposure of residues L20 and L22 towards the TfR and in particular the interaction of TfR-helix1 with Leu 22 [46]. This loop plays a crucial role in the interaction of the two proteins, its substitution results in a ~ 10-fold reduction in affinity for TfR [47]. The second example shows the deformation of residues 100-103, forming a loop at the surface of the transthyretin upon complexation with a molecule of retinol-binding protein ([PDB:1RLB] chain A). It corresponds to the transition from a straight (modeled by *surface-letter *[H]) to a curved conformation (modeled by *core-letter *[O]). It appears that this deformation is due to residue S100 that is pushed towards the protein interior while interacting with the partner, inducing a rotation of P102 (Figure 6C).

#### From regular to irregular local conformations

**Figure 7:**
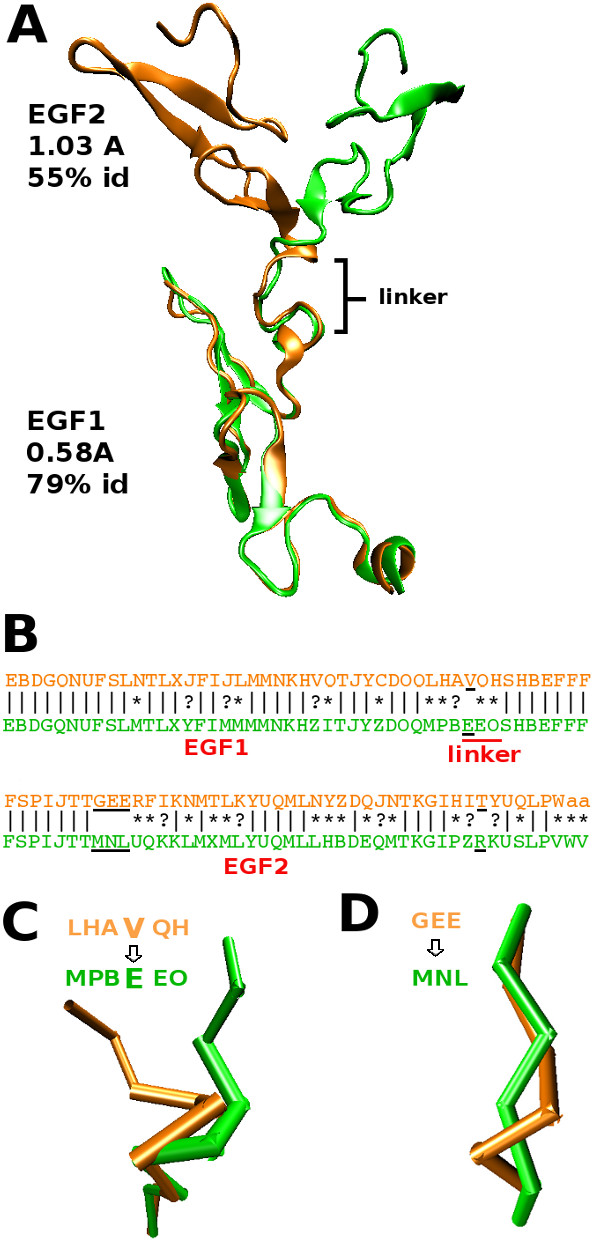
**Deformation between regular and non-regular secondary structures**. A) The structural superimposition of the EGF1 and EGF2 domains of the light chain of the coagulation factor VIIa in unbound (orange) and bound (green) states. B) The alignment of the structural sequences associated with the bound and unbound states of the protein is presented, | stands for identities, * for transitions between structural letters of the same secondary structure type, ? for transitions involving border-letters. The three deformed regions that do not fit the deformation tendencies are underlined in black. B and D) Zoom on the linker region (B) and region close to linker region (D) that undergo deformations that violate the deformation tendencies, the corresponding structural sequences are shown.

The last example regards the light chain of the coagulation factor VIIA (fVIIa) inhibited with a BTPI-mutant ([PDB:1FAK] chains HL,T). Although the overall C*_α _*RMSD between the bound and unbound states indicates a strong deformation upon interaction (3.71Å), the two EFG-like modules (EGF1 and EGF2) are structurally similar with respectively 0.58Å and 1.03Å C*_α _*RMSD and 79% and 55% structural sequence identity. The EGF1 domain rotates ≈ 180° about the linker hexapeptide (positions 85-90) compared to its position in the unbound state thanks to a single change in the main-chain torsion angles of D88 [48,49] (Figure 7A. Among the 37 modified structural letters between the bound and unbound structural sequences associated with the light chain of fVIIa, 30 correspond to deformations that follow the induced-fit modification tendencies: 23 are associated with modifications between letters of the same secondary structure type and 9 involved border-letters previously shown to be the most deformed local conformations upon interaction. The 5 remaining deformed positions are found in three regions with successive changed letters and correspond to changes between letters of different structural type (Figure 7B). The first region ([PDB:1FAK] chain L:82-90) corresponds to the linker region and is associated with a 2.05Å C*_α _*RMSD. It characterizes the conformational modification required for the rotation of the EGF1 module from an helical conformation modified to a loop one "AV"→"BE" (Figure 7C). The second modified region in the structural sequences ([PDB:1FAK] chain L:102-107) indicates a deformation from a loop to a *β*-strand conformation "GEE"→"MNL" (Figure 7D). The proximity of this region to the linker region suggests some broken interactions are responsible for this local deformation (in particular residues D104, I90 and D88). The last modified region ([PDB:1FAK] chain L:132-135) is located in the C-ter of the protein.

The detection of local deformations in the backbone of the proteins by this local approach highlights the importance not only to consider deformation between different secondary structure types but also the conformational variations that occur within the different secondary structure types. While deformation tendencies define general features for secondary structures induced-fit modification coherent with the compartment preference of local conformations, the example cases show more drastic structural modifications that violate the deformation tendencies due to strong structural constraints for functional reasons.

## Conclusions

Descriptors of protein interfaces based on amino acid composition and evolution, structural features and complementarity are fundamental to the understanding, prediction and modeling of protein-protein interactions [5,9,50-52] and ultimately to protein functions. Recent work on ubiquitin has shown the need for efficent structural descriptors able to characterize local conformations [30,32]. Here we use the structural alphabet HMM-SA that allows the identification of local variations in secondary structure conformations. Loops can be characterized despite their high plasticity that inhibits their description by global approaches [53]. The straight or curved shape of regular secondary structures can be detected. Our analysis reveals new structural features, regarding the shape and induced-fit deformation of secondary structures, which have not been appreciated before. In particular, variations in the shape of secondary structures have been analysed thanks to the local approach for the different types of complexes and results are shown to be stable between homodimers, heterodimers, obligate and transient complexes. The large-scale analysis of secondary structure changes in proteins from disordered to ordered secondary structure and between different secondary structure types using a global approach has shown the importance of secondary structure modification for protein function [33]. Here we show that conformational modification within secondary structures can be further analyzed and detailed using to the local approach. We show that the local conformations associated with the different types of secondary structures are not uniformly distributed within proteins at interface, in the core and on the surface, but show compartment preferences that can be related to structural characteristics. In the light of this new structural description of protein compartments, we revisited the induced-fit modifications of local conformation analysis proposed in [34].

The local conformations modeled by the 27 structural letters of HMM-SA are associated with variation in secondary structure conformation. We observed that they present preferential distributions at protein interface, surface and core which affect around 14% of the loop-letters, 23% of the *β*-letters and 3% of the *α*-letters. The greatest difference occurs between protein surface and core, where straight local conformations are preferred in core while curved ones are preferred on surface with the particularity for some of them to be avoided at interface. The proportion of a local conformation at interface is generally intermediate between its proportion on surface and in the core suggesting that interface scaffolds are formed by secondary structures mixing local conformations preferred on surface with ones preferred in the core. Previous analysis on amino acid composition have led to the description of protein-protein interfaces as regions displaying intermediate properties between those of the hydrophilic protein surface and the hydrophobic protein core [40,54], hydrophobic and polar residues are organized in a core/rim interface [6,7]. Local conformations preferentially distributed on the surface tend to be more accessible to solvent at interface than local conformations prefered in the core. This suggests a specific organisation of the local conformations in the binding site (similarly to the amino acids). However the amino acid composition of the local conformations appears to be not correlated with their compartment preference, exposure to solvent of residues is more likely to play a role. Moreover the fact that some local conformations are found to be avoided at interface in both protein bound and unbound states and that local loop conformations are mainly unchanged upon complexation suggests that such organisation is prior to the interaction. Binding sites would be structurally optimized to interact with protein partners. This latter remark is supported by a large-scale analysis of protein-protein interface performed by a global approach showing that favorable interface structural scaffolds have been re-used and adapted by evolution for diverse functions [24]. To the authors' knowledge, the analysis and results presented here have not been reported before and have been elucidated thanks to the use of a local approach able to described the conformation of secondary structures elements in more details than global approaches. These findings should be considered for accurate protein structure reconstruction either based on structural alphabet [55] or on efficient secondary structure conformation prediction [56].

The analysis proposed in [34] has opened the path to an innovative way to analyse structural modifications upon complexation and has highlighted differences between local conformations regarding deformation. By revisiting the induced-fit modifications of local conformations in the light of their compartment preference and structural characteristics, we gain further insight into the deformation properties of local conformations, and of secondary structures to a larger extent, upon protein-protein complex formation. For regular secondary structures, curved conformations (surface preference) tend to be mostly deformed at interface towards straight conformations (core preference), these deformations could be a mechanistic effect of the interaction with the partner leading to a structural adaptive flattening of the interface's surface and a decrease of solvent exposure. For loops, deformation of local conformations appears to be mainly associated with the conservation of the exterior exposure suggesting that loops adopt optimized conformations prior to the interaction. Deformations associated with a modification of the exposure to protein exterior are suggested to favor/unfavor residue interaction with the partner. The low number of this latter type of deformation fits with the fact that only few residues at interface are under strong structural/functional constraints. Interestingly, flanking regions present a different behavior compared to secondary structures being highly deformed. It highlights their important structural adaptive role in the reorganisation of secondary structures between them upon interaction. Induced-fit modification tendencies defined from this analysis should be valuable information to consider for docking tools that aim to consider proteins flexibility [25,57] since protein deformation can be of critical importance for protein interaction. Finaly, we present example cases where the violations of the induced-fit modification tendencies derived from this analysis are associated with strong structural constraints directly related to the function of the proteins. An example illustrates transitions between local conformations associated with different secondary structure types which characterize the deformation of a linker and of a neighboring region involved in the open/closed conformation of the protein. More globally, transitions between different secondary structure types have been shown to play an important role in protein function [58-60] and are observed in a variety of proteins [33]. Therefore the possibility to finely detect and characterize such transitions is an important point of this study. Another example of the violation of the induced-fit modification tendencies is the deformation from straight to curved *α*-helices involved in the inhibitory conformation of a protein. The detection of such subtle deformations by the local approach highlights the importance not only of considering deformations between different secondary structure types but also the conformational variations that occur within them. Such considerations should allow a better understanding of the role of secondary structures in the functional mechanism of proteins.

## Methods

### Datasets of protein-protein complexes

#### Complete dataset

Among the 8205 complexes with different interface scaffold described in [61], we select a set of 1496 two-chain protein complexes (1283 PDB entries) that present i) structure resolution below 2.5Å, ii) R-factor below 0.3 and iii) at least three other two-chain protein complexes in the PDB that share the same structural scaffold at interface. This dataset is constructed to avoid biases owes to similar interface scaffolds between the proteins of the dataset.

#### Homo/heterodimers, transient/obligate complexes

Four other datasets previously described in the literature are used here to distinguish among the different types of protein-protein complexes. These are denoted Homodimers (93 complexes [7]), Heterodimers (203 complexes [41]), Transient and Obligate complexes (70 and 96 complexes respectively [62]) datasets. 49% (respectively 17%) of the PDB entries in the transient complexes (respectively heterocomplexes) dataset are shared with the heterocomplexes (respectively transient complexes) dataset, homodimers and obligates complexes shares less than 5% of PDB entries.

#### Bound/Unbound proteins

Two more additional datasets extracted from the version 2.4 of the benchmark proposed in [63,64] are used: 84 crystallographic structures of transient complexes (bound state) to which are associated the corresponding structures of the free proteins (unbound state).

### Definition of protein compartments: Interface, surface and core

Proteins are divided into three *compartments: interface, surface *and *core*. Residues are assigned to one of the three compartments according to their percentage of relative solvent accessibilities in the disjoint bound conformation (noted *A_chain_*), in the two-chain complex forming the interface of interest (noted *A_interf_*) and in the higher complex considering all chains described in the PDB entry (noted *A_complex_*). *Core residues *correspond to residues *r *that are buried in the core of the protein ($A_{chain}^{r}<5\%$) and whose relative solvent accessibility is not modified when the chain is associated with the other chains of the complex ($A_{chain}^{r}-A_{complex}^{r}=0\%$). These residues constitute the core compartment of proteins. *Surface residues *correspond to residues *r *that are exposed at protein surface ($A_{chain}^{r}>5\%$) and that do not display solvent accessibility variation in the stand-alone chain compared to the higher complex ($A_{chain}^{r}-A_{complex}^{r}=0\%$). These residues constitute the *surface compartment. Interface residues *correspond to residues *r *that are exposed at protein surface ($A_{chain}^{r}>5\%$) and whose relative solvent accessibility is modified when the two chains forming the interface of interest are associated ($A_{chain}^{r}-A_{interf}^{r}>1\%$). These residues constitute the *interface compartment*. Residues that do not fit one of these three definitions are denoted *undefined *and are not considered for the analysis since they cannot be assigned to a compartment. The definition of interface compartments in this work aims to take into account residues affected by the binding of the partner rather that only those which interact with it. This choice is based on previous studies which argued that interaction of protein partners may not only be due to specific interaction of residues but also to non-partner specific structural features surrounding the interacting residues (favorable interface scaffolds [24], convergent local structural motifs [34]). Therefore, similarly to [24] where the interface definition also considers neighboring residues to interacting ones since they provide the interface scaffold, we define as interfacial residues those with 1% solvent accessibility change upon interaction in order to largely consider the residues of the secondary structures forming the interface scaffold.

### Residues and structural letters

The 3 D structures are described as series of overlapping four-residues fragments modeled by a structural letter. Therefore a residue *r *is associated with four different fragments *L*_1_, ..., *L*_4 _where *L*_1 _corresponds to the four successive residues *r *- 3 → *r *and *L*_4 _to the four successive residues *r *→ *r *+ 3. Each four-residue fragment is associated with a structural letter describing its conformation, a protein structure of *N *residues is encoded in a sequence of *N *- 3 structural letters. The physico-chemical characteristics and the compartment assignment of the structural letter encoding the fragment *r *- 2 → *r *+ 1 are determined according to the properties of the residue *r *as in [34].

### Qualitative statistical analysis

#### Multiple Correspondence Analysis

Multiple Correspondence Analysis (MCA) is a qualitative multivariate method used here for the 2 D representation of the structural letters' occurrence in each of the three protein compartments [65]. The graphical display of the MCA allows the qualitative analysis of the structural letters' preference for proteins interface, surface or core compartments.

#### Principal Component Analysis

Principal Component Analysis (PCA) is a multivariate method used here for the representation of the structural descriptors of the structural letters. The PCA transforms the variables into a smaller number of uncorrelated variables (principal components) [66].

### Quantitative statistical analysis

#### Kullback-Leibler measure

The non-symmetrized Kullback-Leibler divergence measure (KLd) is a statistical criterion used here to assess the asymmetrical distribution of the structural letters in the three compartments, taking into account the secondary structural type of the letters. The KLd is computed as follows:

$$KLd(sl)=\;\sum_{cp=1}^{3}Psl,cp\times ln\left(\frac{Psl,cp}{Pss,cp}\right)$$

where *cp *is a compartment, *sl *is a given structural letter, *ss *is the set of letters of the same secondary structure type than *sl, p_sl,cp _*is the frequency of *sl *in compartment *cp *(i.e. occurence of *sl *in *cp *over *N_sl _*the occurence of *sl *in the 3 compartment) and *p_ss,cp _*is the frequency of *ss *in compartment *cp *(i.e. occurence of *ss *in *cp *over the occurence of *ss *in the three compartment). The KLd values can be assessed by a *χ*^2 ^test, since the quantity 2*N_sl _*× *KLd*(*sl*) (denoted KLd quantities) follows a *χ*^2 ^distribution.

#### Z-score computation

Z-scores are computed to assess the preferred compartment of a structural letter:

$$Z_{cp1/cp2}(sl)=\frac{N_{cp1}^{obs}(sl)-N_{cp1}^{exp_{cp1/cp2}}(sl)\;}{\sqrt{N_{cp1}^{exp_{cp1/cp2}}(sl)}}$$

where *sl *is a given structural letter, $N_{cp1}^{obs}(sl)$ is the observed occurrence of *sl *in compartment *cp*1, $N_{cp1}^{exp_{cp1/cp2}}(sl)$ is the expected occurrence of *sl *in compartment *cp*1 if distributions in *cp*1 and *cp*2 were similar. $N_{cp1}^{exp_{cp1/cp2}}(sl)$ = *N*_*cp*1_(*sl*) × *f*_*cp*2_(*sl*) where *N*_*cp*1_(*sl*) is the occurrence of *sl *in *cp*1 and *f*_*cp*2_(*sl*) the relative frequency of *sl *in *cp*2. $N_{cp1}^{exp}(sl)$ has to be > 5 for the Z-score to be statistically meaningful. A Bonferoni correction is applied on each test to determine the significativity threshold *T *: *Z*_*cp*1/*cp*2_(*sl*) *> T *indicates a significant preference of *sl *for compartment *cp*1, *Z*_*cp*1/*cp*2_(*sl*) *<*-*T *indicates a significant preference for *cp*2.

### Relative solvent accessibility calculation

Relative solvent accessibilities of residues are calculated using NACCESS 2.1.1 [67] with a probe size of 1.4Å. Relative accessibilities are calculated for each residue in a protein by expressing the summed residue accessible surfaces as a percentage of that observed in a ALA-X-ALA tripeptide built using the QUANTA molecular graphics package in extended conformations.

### Quantification of structural letters deformation at interface

In order to evaluate the conformational changes of secondary structures upon interaction, the deformation of local conformations is analysed by comparing the substitution of the structural letters from the unbound to the bound state using *P *(*sl*_1_, *sl*_2_), that is the number of letter *sl*_1 _deformed in letter *sl*_2 _over the total number of letter *sl*_1 _deformed upon interaction. Notice that differences due to deformation rate difference among the letters are avoided by only considering deformed letters. Since the natural flexibility of proteins should lead to similar structural letter substitutions at interface and surface, we focused on the deformation of local conformations induced by complex formation that occurs at interface by computing the following quantities:

$$\Delta P(sl_{1},sl_{2})=P_{interf}(sl_{1},sl_{2})-P_{surf}(sl_{1},sl_{2})$$

where *P_inter f _*(*sl*_1_, *sl*_2_) is calculated for letters at protein interface and *P_sur f _*(*sl*_1_, *sl*_2_) for letters at protein surface. The idea here is that deformations which differ the most between interface and surface (Δ*P *(*sl*_1_, *sl*_2_) >> 0) are more likely to be induced by the interaction.

## Authors' contributions

JB carried out the analysis. ACC and JB conceived the study. All authors read and approved the final manuscript.

## Supplementary Material

Additional file 1 **Structural descriptors of the 27 structural letters**. Structural letters are associated with specific conformations of four consecutive residues described by four descriptor: *d*_1 _(distance between the *α*-carbons of residues 1 and 3), *d*_2 _(distance for residues 1 and 4), *d*_3 _(distance for residues 2 and 4) and *P*_4 _(the oriented projection of the last *α*-carbon to the plane formed by the three first ones). Click here for file

Additional file 2 **Residues distribution in the protein compartments**. For each dataset, the total number of residues (*N*) is given, as well as the proportion of residues at interface (%), surface (%), core (%) and the proportion of residues which do not fit the definition of one of the three compartments (Undef%). Click here for file

Additional file 3 **Multiple correspondence analysis performed on loop- and border-letters for homodimers, heterodimers, obligate, transient complexes and protein chains in bound and unbound states**. The first axis differentiates the surface-letters from the core-letters. Letters are similarly distributed around this axis for all the different datasets. The second axis differentiates interface from non-interface region, variations along this second axis are observed for the different letters according to the dataset, excepted for letter [D] prefered in non-interface region and letter [F] preferred in interface region Click here for file

Additional file 4 **MCA performed on β-letters for homodimers, heterodimers, obligate, transient complexes and protein chains in bound and unbound states**. The first axis differenciates the surface-letters from the core-letters. Letters are similarly distributed around this axis for all the different datasets. Particularly, letters [L] and [N] are clearly associated with the surface and the non-interface region in the all seven datasets while [M] is associated with the core. The MCA plot obtained for transient complexes shows a difference for letter [T] which appears to be preferred in the non-interface region in opposite to its tendency to prefer interface in homodimers, heterodimers and obligate complexes. This contradictive behavior is less pronounced in the bound and unbound dataset. Click here for file

Additional file 5 **MCA performed on α-letters for homodimers, heterodimers, obligate, transient complexes and protein chains in bound and unbound states**. Preferences of *α*-letters among the seven datasets are less stable than for the other structural letters. This agrees with other analysis of this study where *α*-letters display the weaker distribution signal and the most similar structural properties among them. Globally the first axis tends to differentiate between surface and core excepted for obligate complexes where it differentiates between interface and non-interface regions. However, the behavior of the two letters [a] and [A] are stable among the different datasets being preferentially distributed in non-interface region and in core respectively. Click here for file

Additional file 6 **Detailed evaluation of the percentage of secondary structures affected by the preferential distribution in the complete dataset**. Counting of structural letters at interface, surface and core in the complete dataset. The observed (Obs) and expected (Exp) numbers of structural letters at interface, surface and core are given and the difference between the two is calculated (Diff). For each structural type, the sum of the difference is calculated to evaluate the proportion of the secondary structure affected by the preferential distribution. Click here for file

Additional file 7 **Amino acid composition of the structural letters associated with regular secondary structures**. Amino acid composition at interface (white), on surface surface (grey) and in core (black) for *α*-letters [a,A,V,W], *β*-letters [L,M,N,T,X] and border-letters [B,C,Z,K,J]. No common amino acid specificities are observed between letters associated with identical compartment. Click here for file

Additional file 8 **Amino acid composition of the structural letters associated with loops**. Amino acid composition at interface (white), on surface (grey) and in core (black) for loop-letters. Surface letters [P,H,Y] present high proportion of proline and a small proportion of glycine and therefore present a similar amino acid composition profile to core-letter [R] than to surface-letter [U]. Interface-letter [F] present a high proportion of both residues glycine and proline while non-interface-letter [D] appears to be particularly enriched in glycine. Click here for file

Additional file 9 **Deformation matrices for surface and core compartments**. Proportion matrix *P *(*ω, ψ*) where *ω *is the letter in the unbound state (y-axis) and *ψ *the corresponding letter in the bound state (x-axis). Structural letters are separated according to their structural type with black lines, and differentiated according to their compartment preferences (blue for core, red for surface, triangle for interface and square for non interface). Grey dotted lines separated surface loop-letters from core ones. Click here for file

## References

[B1] Ofran Y Rost B Analysing six types of protein-protein interfaces J Mol Biol 2003 325 377 387 10.1016/S0022-2836(02)01223-8 12488102

[B2] Lo Conte L Chothia C Janin J The atomic structure of protein-protein recognition sites J Mol Biol 1999 285 2177 2198 10.1006/jmbi.1998.2439 9925793

[B3] Glaser F Steinberg D Vakser I Ben-Tal N Residue frequencies and pairing preference at protein-protein interfaces Proteins 2001 43 89 102 10.1002/1097-0134(20010501)43:2<89::AID-PROT1021>3.0.CO;2-H 11276079

[B4] Res I Lichtarge O Character and evolution of protein-protein interfaces Phys Biol 2005 2 S36 S43 10.1088/1478-3975/2/2/S04 16204847

[B5] Guharoy M Chakrabarti P Conserved residue clusters at protein-protein interfaces and their use in binding site identification BMC Bioinformatics 2010 11 286 10.1186/1471-2105-11-286 20507585 PMC2894039

[B6] Chakrabarti P Janin J Dissecting protein-protein recognition sites Proteins 2002 15 334 343 10.1002/prot.10085 11948787

[B7] Bahadur R Chakrabarti P Rodiffer F Janin J Dissecting subunit interfaces in homodimeric proteins Proteins 2003 53 708 719 10.1002/prot.10461 14579361

[B8] Neuvirth H Raz R Schreiber G ProMate: a structure based prediction program to identify the location of protein-protein binding site J Mol Biol 2004 338 181 199 10.1016/j.jmb.2004.02.040 15050833

[B9] Hoskins J Lovell S Blundell T An algorithm for predicting interaction sites: abnormally exposed amino acid residues and secondary structure elements Protein Sci 2006 5 1017 1029 10.1110/ps.051589106 PMC2242518 16641487

[B10] Guharoy M Chakrabarti P Secondary structures based analysis and classification of biological interfaces: identification of binding motifs in protein-protein interactions Bioinformatics 2007 23 1909 1918 10.1093/bioinformatics/btm274 17510165

[B11] Betts M Sternberg M An analysis of conformational changes on protein-protein association: implications for predictive docking Protein Eng 1999 12 271 283 10.1093/protein/12.4.271 10325397

[B12] Smith G Sternberg M Bates P The relationship between the flexibility of proteins and their conformational states on forming protein-protein complexes with an application to protein-protein docking J Mol Biol 2005 347 1077 1101 10.1016/j.jmb.2005.01.058 15784265

[B13] Yogurtcu O Erdemli S Nussinov R Turkay M Keskin O Restricted mobility of conserved residues in protein-protein interfaces in molecular simulations Biophys J 2008 94 3475 3485 10.1529/biophysj.107.114835 18227135 PMC2292389

[B14] Valdar W Thornton J Conservation helps to identify biologically relevant crystal contacts J Mol Biol 2001 313 399 416 10.1006/jmbi.2001.5034 11800565

[B15] Mintseris J Weng Z Atomic contacts vectors in protein-protein recognition Proteins 2003 53 629 639 10.1002/prot.10432 14579354

[B16] Jeerson E Walsh T Barton G Biological units and their effects upon the properties and prediction of protein-protein interactions J Mol Biol 2006 364 1118 1129 10.1016/j.jmb.2006.09.042 17049359

[B17] De S Krishnadev O Srinivasan N Rekha N Interaction preferences across protein-protein interfaces of obligatory and non-obligatory components are different BMC Struct Biol 2005 16 15 10.1186/1472-6807-5-15 PMC1201154 16105176

[B18] Zhanhua C Gah-Kok Gan J Lei L Sakharkar M Kangueane P Protein subunit interfaces: heterodimers versus homodimers Bioinformation 2005 2 28 39 10.6026/97320630001028 PMC1891636 17597849

[B19] Mintseris J Weng Z Structure, function and evolution of transient and obligate protein-protein interactions Proc Natl Acad Sci 2005 102 10930 10935 10.1073/pnas.0502667102 16043700 PMC1182425

[B20] Vacic V Uversky V Dunker A Lonardi S Composition Profiler: a tool for discovery and visualization of amino acid composition difference BMC Bioinformatics 2007 8 211 10.1186/1471-2105-8-211 17578581 PMC1914087

[B21] Jones S Thornton J Protein-protein interactions: a review of protein dimer structures Prog Biophys Molec Biol 1995 63 31 65 10.1016/0079-6107(94)00008-W 7746868

[B22] Argos P An investigation of protein subunit and domain interfaces Protein Eng 1998 2 101 113 10.1093/protein/2.2.101 3244692

[B23] Miller S The structure of interfaces between subunits of dimeric and tetrameric proteins Protein Eng 1989 3 77 83 10.1093/protein/3.2.77 2594726

[B24] Keskin O Nussinov R Favorable scaffolds: proteins with different sequence, structure and function may associate in similar ways PEDS 2005 18 11 24 15790576 10.1093/protein/gzh095

[B25] May A Zacharias M Accounting for global protein deformability during protein-protein and protein-ligand docking Biochim Biophys Acta 2005 30 225 231 10.1016/j.bbapap.2005.07.045 16214429

[B26] Koshland D Application of a theory of enzyme specificity to protein synthesis Proc Natl Acad Sci 1958 44 98 104 10.1073/pnas.44.2.98 16590179 PMC335371

[B27] Tsai C Kumar S Ma B Nussinov R Folding funnels, binding funnels and protein function Protein Sci 1999 8 1181 1190 10.1110/ps.8.6.1181 10386868 PMC2144348

[B28] Daily M Gray J Local motions in a benchmark of allosteric proteins Proteins 2007 67 385 399 10.1002/prot.21300 17295319

[B29] Goh CS Milburn D Gerstein M Conformational changes associated with protein-protein interactions Curr Op Struct Biol 2004 14 104 109 10.1016/j.sbi.2004.01.005 15102456

[B30] Wlodarski T Zagrovic B Conformational selelction and induced fit mechanism underlie specifity in non-covalent interactions with ubiquitin Proc Natl Acad Sci 2009 106 19346 19351 10.1073/pnas.0906966106 19887638 PMC2780739

[B31] Gutteridge A Thornton J Conformational changes observed in enzyme crystal structures upon substrate binding J Mol Biol 2005 346 21 28 10.1016/j.jmb.2004.11.013 15663924

[B32] Perica T Chothia C Ubiquitin - molecular dynamics for recognition of different structures Curr Op Struct Bio 2010 20 367 376 10.1016/j.sbi.2010.03.007 20456943

[B33] Dan A Ofran Y Kliger Y Large-scale analysis of secondary structure changes in proteins suggests a role for disorder-to-order transitions in nucleotide binding proteins Proteins 2009 78 236 248 10.1002/prot.22531 19676113

[B34] Martin J Regad L Lecornet H Camproux A Structural deformation upon protein-protein interaction: a structural alphabet approach BMC Struct Biol 2008 18 12 10.1186/1472-6807-8-12 PMC2315654 18307769

[B35] Kumar S Bansal M Geometrical and sequence characteristics of alpha-helices in globular proteins Biophys 1998 75 1935 1944 10.1016/S0006-3495(98)77634-9 PMC1299864 9746534

[B36] Camproux A Gauthier R Tuery P A hidden Markov model derived structural alphabet for proteins J Mol Biol 2004 339 591 605 10.1016/j.jmb.2004.04.005 15147844

[B37] Camproux A Tuffery P Hidden Markov Model-derived structural alphabet for proteins: the learning of protein local shapes captures sequence specificity Biochim Biophys Acta 2005 1724 394 403 16040198 10.1016/j.bbagen.2005.05.019

[B38] Regad L Martin J Camproux A Identification of non-random motifs in loops using a structural alphabet IEEE Symposium on Computational Intelligence and Bioinformatics and Computational Biology 2006 1 9

[B39] Miller S Janin J Lesk A Chothia C Interior and surface of monomeric proteins J Mol Biol 1987 196 641 656 10.1016/0022-2836(87)90038-6 3681970

[B40] Jones S Thornton J Principles of protein-protein interactions Proc Natl Acad Sci 1996 93 13 20 10.1073/pnas.93.1.13 8552589 PMC40170

[B41] Pal A Chakrabarti P Bahadur R Rodiffer F Janin J Peptide segments in protein-protein interfaces J Biosci 2007 32 101 111 10.1007/s12038-007-0010-7 17426384

[B42] Bogan A Thron K Anatomy of hot spots in protein interfaces J Mol Biol 1998 280 1 9 10.1006/jmbi.1998.1843 9653027

[B43] Kawabata T MATRAS: a program for protein 3 D structure comparison Nuc Ac Res 2003 31 3367 3369 10.1093/nar/gkg581 PMC168987 12824329

[B44] Huse M Chen YG Massague J Kuriyan J Crystal structure of the cytoplasmic domain of the type I TGF-beta receptor in complex with FKBP12 Cell 1999 96 425 436 10.1016/S0092-8674(00)80555-3 10025408

[B45] Huse M Muir T Chen YG Kuriyan J Massague J The TGF-beta receptor activation process: an inhibitor- to substrate-binding switch Molecular Cell 2001 8 671 682 10.1016/S1097-2765(01)00332-X 11583628

[B46] Bennett M Lebron J Bjorkman P Crystal structure of the hereditary haemochromatosis protein HFR complexed with transferin receptor Nature 2000 403 46 53 10.1038/47417 10638746

[B47] Lebron J Bjorkman P The transferrin receptor binding site on HFE, the class I MHC-related protein mutated in hereditary hemochromatosis J Mol Biol 1999 289 1109 1118 10.1006/jmbi.1999.2842 10369785

[B48] Pike A Brzozowski A Roberts S Olsen O Persson E Structure of human factor VIIa and its implications for the trigerring of blood coagulation Proc Natl Acad Sci 1999 96 8925 8930 10.1073/pnas.96.16.8925 10430872 PMC17709

[B49] Zhang E Charles RS Tulinsky A Structure of extracellular tissue factor complexed with factor VIIa inhibited with a BTPi mutant J Mol Biol 1999 285 2089 2104 10.1006/jmbi.1998.2452 9925787

[B50] Ban Y Edelsbrunner H Rudolph J Interface surfaces for protein-protein complexe J ACM 2006 53 361 378 10.1145/1147954.1147957

[B51] Darnell S Page D Mitchell J An automated decision-tree approach to predicting protein interaction hot spots Proteins 2007 68 813 823 10.1002/prot.21474 17554779

[B52] Yu J Guo M Prediction of protein-protein interactions from secondary structures in binding motifs using the statistic method In Proceedings of the 2008 Fourth International Conference on Natural Computation 2008

[B53] Regad L Martin J Nuel G Camproux A Mining protein loops using a structural alphabet and statistical exceptionality BMC Bioinformatics 2010 11 75 10.1186/1471-2105-11-75 20132552 PMC2833150

[B54] Korn A Burnett R Distribution and complementarity of hydropathy in multisubunit proteins Proteins 1991 9 37 55 10.1002/prot.340090106 2017435

[B55] Tuery P Guyon F P D Improved greedy algorithm for protein structure reconstruction J Comput Chem 2005 26 506 513 10.1002/jcc.20181 15693017

[B56] Podtelezhnikov AD Wild D Reconstruction and stability of secondary structure elements in the context of protein structure prediction Biophys J 2009 96 4399 4408 10.1016/j.bpj.2009.02.057 19486664 PMC2711490

[B57] B-Rao C Subramaniana J Sharmaa S Managing protein flexibility in docking and its applications Drug Discovery Today 2009 14 394 400 10.1016/j.drudis.2009.01.003 19185058

[B58] Kim Y Rose C Liu Y Ozaki Y Datta G Tu A FT-IR and near-infrared FT-Raman studies of the secondary structure of insulinotropin in the solid state: alpha-helix to beta-sheet conversion induced by phenol and/or by high shear force J Pharm Sci 1994 83 1175 1180 10.1002/jps.2600830819 7983604

[B59] Jiao W Qian M Li P Zhao L Chang Z The essential role of the flexible termini in the temperature-responsiveness of the oligomeric state and chaperone-like activity for the polydisperse small heat shock protein IbpB from Escherichia coli J Mol Biol 2005 347 871 884 10.1016/j.jmb.2005.01.029 15769476

[B60] Guo J Jaromczyk J Xu Y Analysis of chameleon sequences and their implications in biological processes Proteins 2007 67 548 558 10.1002/prot.21285 17299764

[B61] Tuncbag N Gursoy A Guney E Nussinov R Keskin O Architectures and functional coverage of protein-protein interfaces J Mol Biol 2008 381 785 802 10.1016/j.jmb.2008.04.071 18620705 PMC2605427

[B62] Teyra J Pisabarro M Characterization of interfacial solvent in protein complexes and contributions of wet spots to the interface description J Proteins 2007 67 1087 1095 10.1002/prot.21394 17397062

[B63] Mintseris J Wieke K Pierce B Anderson R Chen R Janin J Weng Z Protein-protein docking benchmarck 2.0: an update. Proteins 2005 60 214 216 10.1002/prot.20560 15981264

[B64] Hwang H Pierce B Mintseris J Janin J Weng Z Protein-protein docking benchmark version 3.0 Proteins 2008 73 705 709 10.1002/prot.22106 18491384 PMC2726780

[B65] Le Roux B Rouanet H Geometric Data Analysis, From Correspondence Analysis to Structured Data Analysis 2004 Dordrecht: Kluwer

[B66] Jolliffe I Principal Component Analysis, Springer Series in Statistics 2002 2 New York: Springer

[B67] Hubbard SJTJ NACCESS Tech. rep., Computer Program, Department of Biochemistry and Molecular Biology, University College London 1993 http://www.bioinf.manchester.ac.uk/naccess/

