# Supplementary material for: Deciphering the shape and deformation of secondary structures through local conformation analysis
Source: BMC Struct Biol. 2011 Feb 1;11:9. doi: 10.1186/1472-6807-11-9 (PMC3224362; doi:10.1186/1472-6807-11-9)
Supplement: Additional file 1 — Structural descriptors of the 27 structural letters. Structural letters are associated with specific conformations of four consecutive residues described by four descriptor: d1 (distance between the α-carbons of residues 1 and 3), d2 (distance for residues 1 and 4), d3 (distance for residues 2 and 4) and P4 (the oriented projection of the last α-carbon to the plane formed by the three first ones). [file 1472-6807-11-9-S1.PDF]

structural letter	d_1	d_2	d_3	P_4
	α-letters			
a	5.39	5.09	5.38	2.92
V	5.41	5.23	5.61	2.86
A	5.43	5.09	5.42	2.94
W	5.62	5.25	5.42	2.87
	β-letters			
L	6.71	9.64	6.50	-2.60
N	6.39	9.93	6.75	-1.07
M	6.87	10.06	6.51	-1.41
T	6.48	10.17	7.09	0.66
X	6.80	10.35	6.85	-0.25
	loop-letters			
D	5.55	7.74	5.60	-3.31
F	6.03	6.85	5.64	-0.63
U	6.47	5.92	5.56	0.53
P	6.57	8.96	5.58	-2.19
H	6.71	8.27	5.47	-3.56
Y	6.87	8.28	6.03	-3.44
E	5.60	6.71	5.50	3.69
O	5.69	8.09	5.67	3.09
S	5.66	8.95	6.54	2.09
R	5.66	8.91	6.66	-1.46
Q	5.66	8.07	6.70	2.96
I	5.70	7.26	7.02	0.88
G	6.21	9.21	5.77	0.27
	border-letters			
Z	5.59	5.49	5.78	2.58
B	5.40	5.58	5.42	3.39
C	5.78	5.68	6.07	1.46
J	6.89	8.94	6.76	-0.48
K	6.72	9.12	6.41	-3.31
