# Supplementary material for: Deciphering the shape and deformation of secondary structures through local conformation analysis
Source: BMC Struct Biol. 2011 Feb 1;11:9. doi: 10.1186/1472-6807-11-9 (PMC3224362; doi:10.1186/1472-6807-11-9)
Supplement: Additional file 2 — Residues distribution in the protein compartments. For each dataset, the total number of residues (N) is given, as well as the proportion of residues at interface (%), surface (%), core (%) and the proportion of residues which do not fit the definition of one of the three compartments (Undef%). [file 1472-6807-11-9-S2.PDF]

N	Interface%	Surface%	Core%	Undef%
Complete dataset				
713,769	12.4%	40.5%	24.8%	22.1%
Homodimers dataset				
55,113	15.8%	42.8%	25.9%	15.3%
Heterodimers dataset				
100,621	18.8%	45.7%	20.7%	14.7%
Obligate dataset				
57,740	15.1%	41.9%	27.2%	15.7%
Transient dataset				
28,991	10.4%	50.0%	24.2%	14.9%
Bound/Unbound dataset				
45,484	8.3%	46.3%	22.4%	23.0%
