# Supplementary figures and images for: Deciphering the shape and deformation of secondary structures through local conformation analysis
Source: BMC Struct Biol. 2011 Feb 1;11:9. doi: 10.1186/1472-6807-11-9 (PMC3224362; doi:10.1186/1472-6807-11-9)

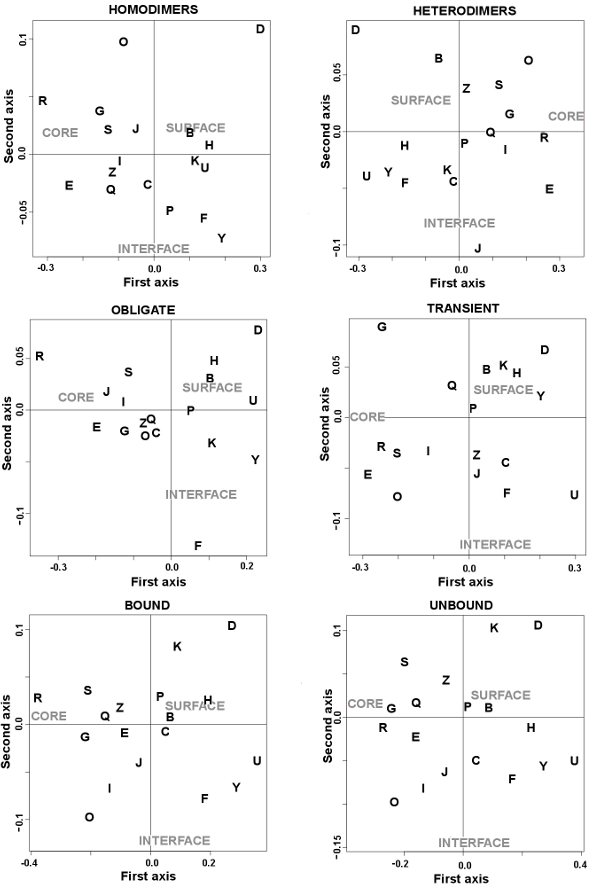

Supplement: Additional file 3 — Multiple correspondence analysis performed on loop- and border-letters for homodimers, heterodimers, obligate, transient complexes and protein chains in bound and unbound states. The first axis differentiates the surface-letters from the core-letters. Letters are similarly distributed around this axis for all the different datasets. The second axis differentiates interface from non-interface region, variations along this second axis are observed for the different letters according to the dataset, excepted for letter [D] prefered in non-interface region and letter [F] preferred in interface region [file 1472-6807-11-9-S3.TIFF]

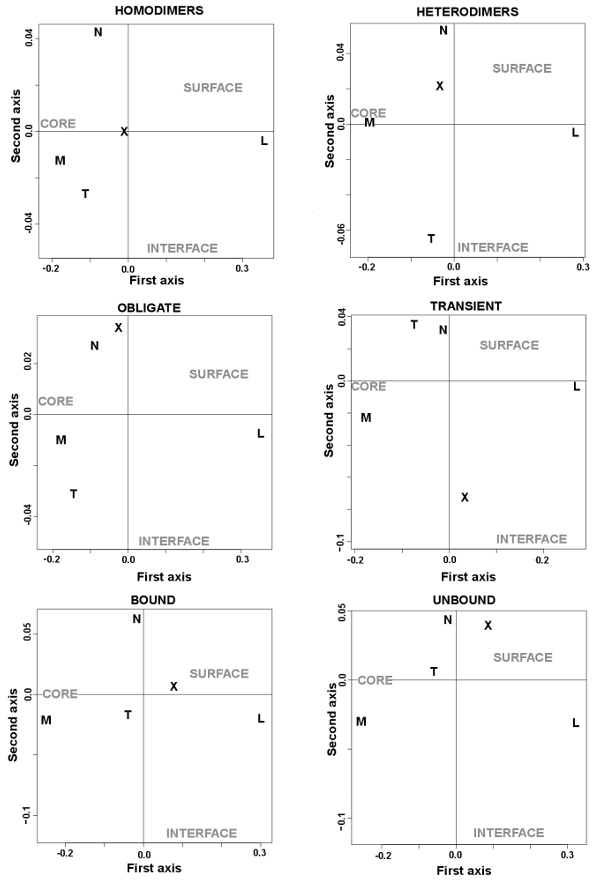

Supplement: Additional file 4 — MCA performed on β-letters for homodimers, heterodimers, obligate, transient complexes and protein chains in bound and unbound states. The first axis differenciates the surface-letters from the core-letters. Letters are similarly distributed around this axis for all the different datasets. Particularly, letters [L] and [N] are clearly associated with the surface and the non-interface region in the all seven datasets while [M] is associated with the core. The MCA plot obtained for transient complexes shows a difference for letter [T] which appears to be preferred in the non-interface region in opposite to its tendency to prefer interface in homodimers, heterodimers and obligate complexes. This contradictive behavior is less pronounced in the bound and unbound dataset. [file 1472-6807-11-9-S4.TIFF]

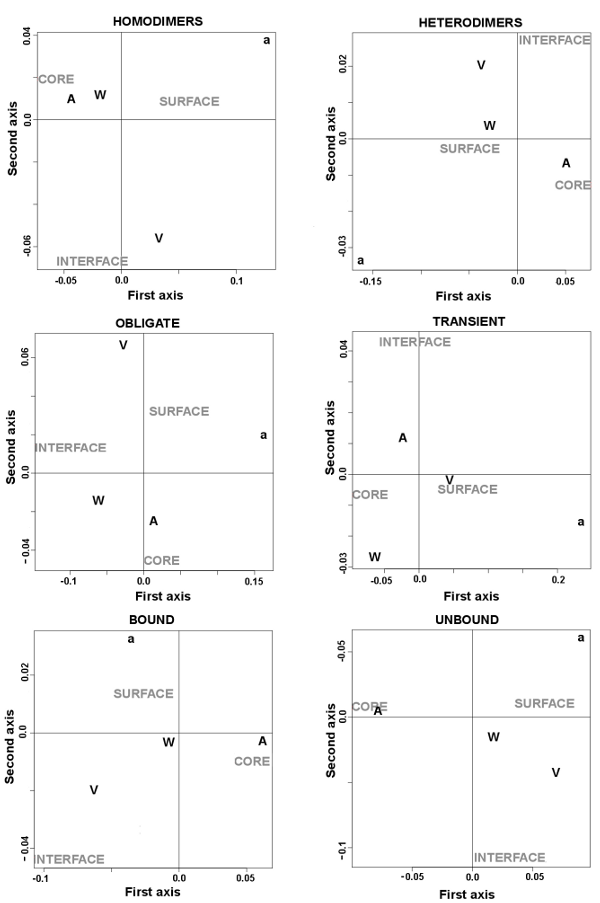

Supplement: Additional file 5 — MCA performed on α-letters for homodimers, heterodimers, obligate, transient complexes and protein chains in bound and unbound states. Preferences of α-letters among the seven datasets are less stable than for the other structural letters. This agrees with other analysis of this study where α-letters display the weaker distribution signal and the most similar structural properties among them. Globally the first axis tends to differentiate between surface and core excepted for obligate complexes where it differentiates between interface and non-interface regions. However, the behavior of the two letters [a] and [A] are stable among the different datasets being preferentially distributed in non-interface region and in core respectively. [file 1472-6807-11-9-S5.TIFF]

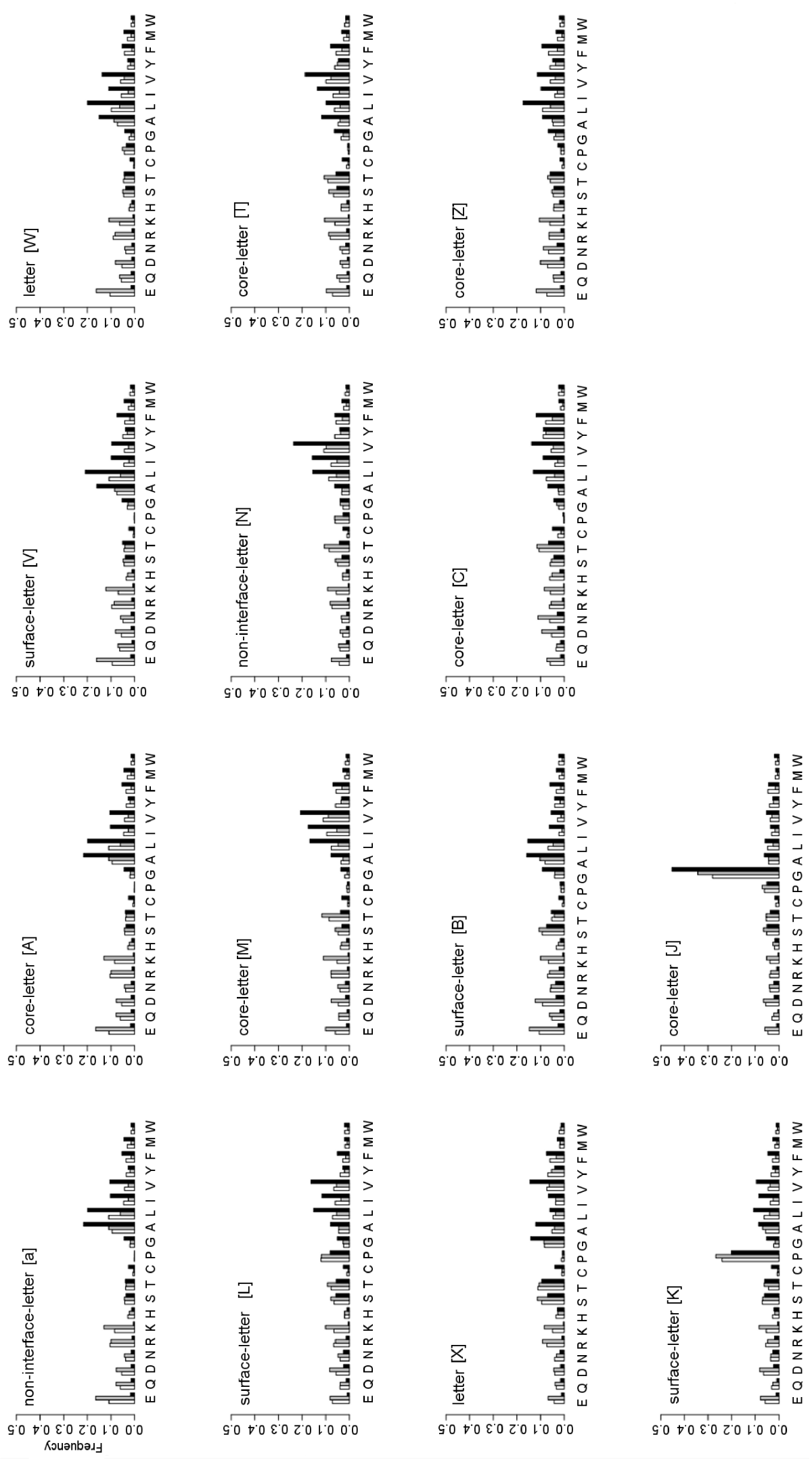

Supplement: Additional file 7 — Amino acid composition of the structural letters associated with regular secondary structures. Amino acid composition at interface (white), on surface surface (grey) and in core (black) for α-letters [a,A,V,W], β-letters [L,M,N,T,X] and border-letters [B,C,Z,K,J]. No common amino acid specificities are observed between letters associated with identical compartment. [file 1472-6807-11-9-S7.TIFF]

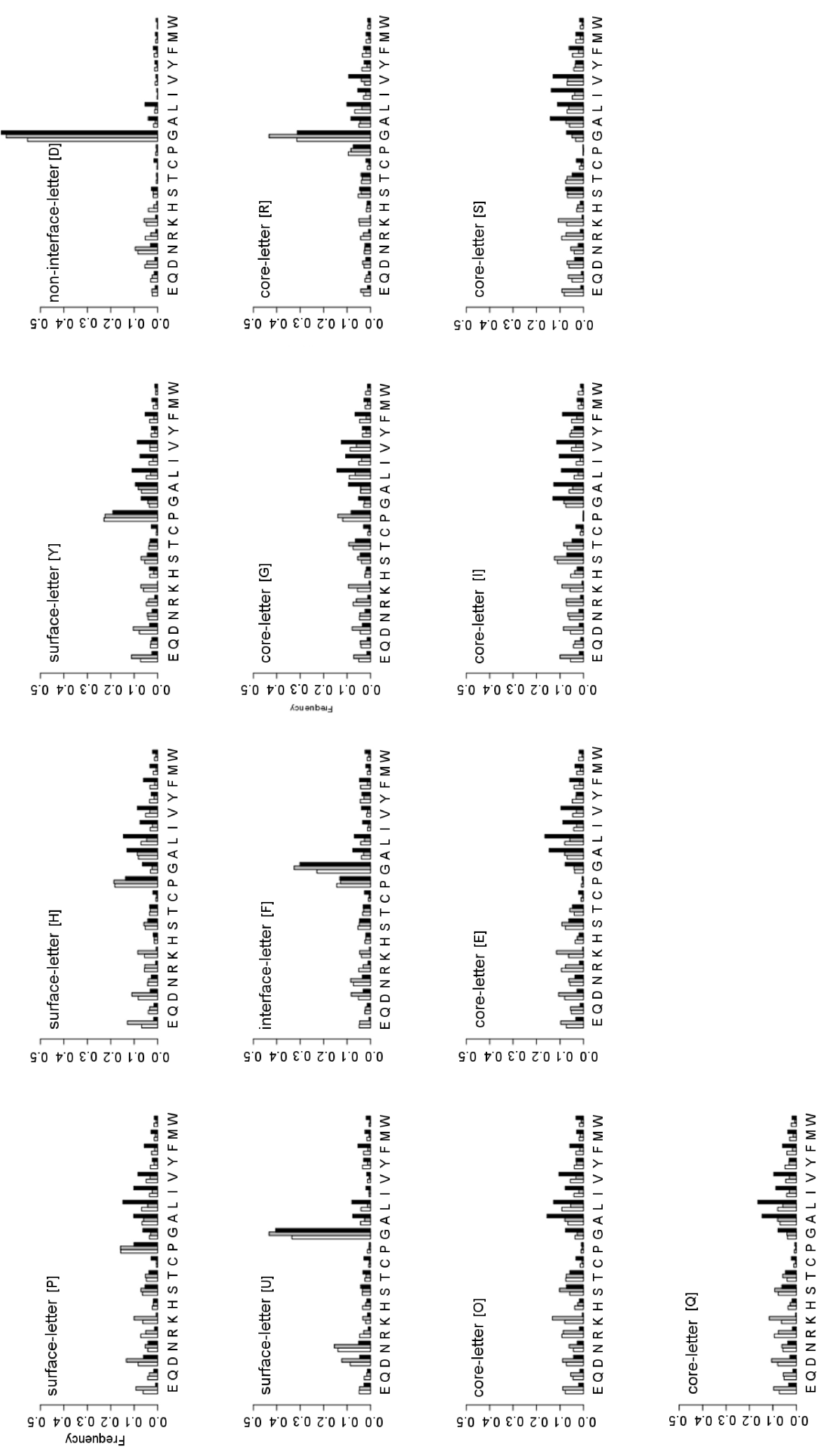

Supplement: Additional file 8 — Amino acid composition of the structural letters associated with loops. Amino acid composition at interface (white), on surface (grey) and in core (black) for loop-letters. Surface letters [P,H,Y] present high proportion of proline and a small proportion of glycine and therefore present a similar amino acid composition profile to core-letter [R] than to surface-letter [U]. Interface-letter [F] present a high proportion of both residues glycine and proline while non-interface-letter [D] appears to be particularly enriched in glycine. [file 1472-6807-11-9-S8.TIFF]

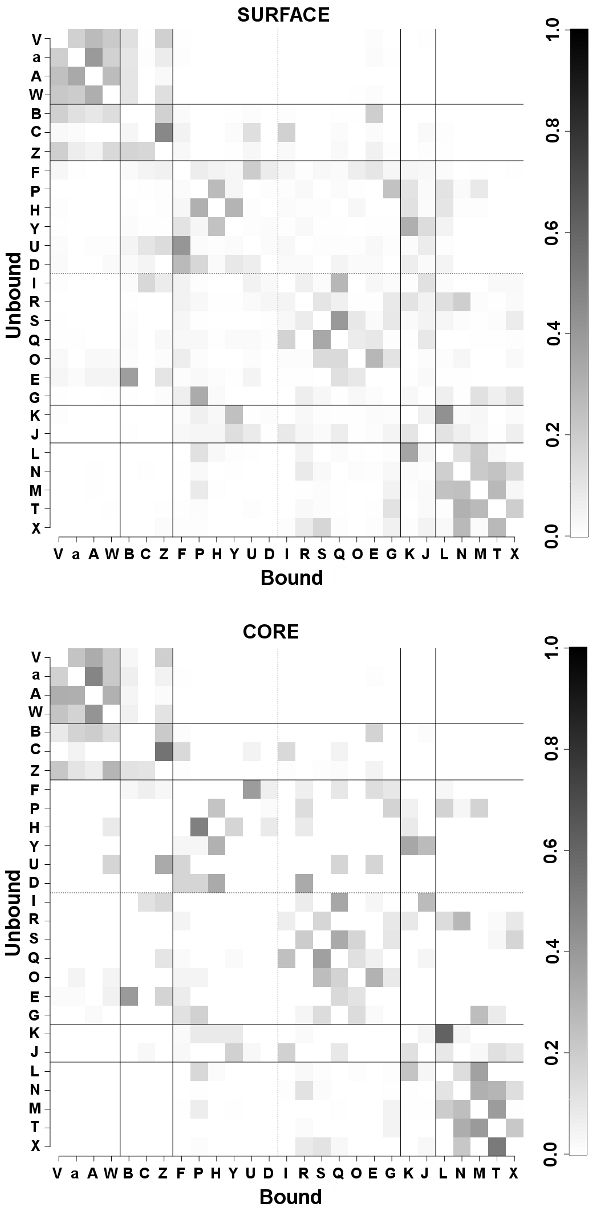

Supplement: Additional file 9 — Deformation matrices for surface and core compartments. Proportion matrix P (ω, ψ) where ω is the letter in the unbound state (y-axis) and ψ the corresponding letter in the bound state (x-axis). Structural letters are separated according to their structural type with black lines, and differentiated according to their compartment preferences (blue for core, red for surface, triangle for interface and square for non interface). Grey dotted lines separated surface loop-letters from core ones. [file 1472-6807-11-9-S9.TIFF]
