# Supplementary material for: Deciphering the shape and deformation of secondary structures through local conformation analysis
Source: BMC Struct Biol. 2011 Feb 1;11:9. doi: 10.1186/1472-6807-11-9 (PMC3224362; doi:10.1186/1472-6807-11-9)
Supplement: Additional file 6 — Detailed evaluation of the percentage of secondary structures affected by the preferential distribution in the complete dataset. Counting of structural letters at interface, surface and core in the complete dataset. The observed (Obs) and expected (Exp) numbers of structural letters at interface, surface and core are given and the difference between the two is calculated (Diff). For each structural type, the sum of the difference is calculated to evaluate the proportion of the secondary structure affected by the preferential distribution. [file 1472-6807-11-9-S6.PDF]

	Interface			Surface			Core			Total
	Obs	Exp	Diff	Obs	Exp	Diff	Obs	Exp	Diff	
a	1337	1491.8	154.8	5499	5056.5	442.4	3600	3887.6	287.6	885 (8%)
V	4771	4657.4	113.5	16520	15786.3	733.6	11290	12137.2	847.2	1694 (5%)
W	4548	4423.1	124.8	14880	14992.1	112.1	11514	11526.6	12.6	249 (0.8%)
A	11639	11722.5	83.5	38669	39733.0	1064.0	31696	30548.4	1147.5	2295 (3%)
Total	477 (2.1%)			2352 (3.1%)			2295 (3.9%)			5124 (3%)
L	4355	3650.7	704.2	14551	10200.0	4350.9	8135	13190.2	5055.2	10110 (37%)
N	3042	3415.4	373.4	8911	9542.5	631.5	13345	12339.9	1005.0	2010 (8%)
M	3606	4025.5	419.5	8895	11247.1	2352.1	17316	14544.3	2771.6	5543 (18%)
T	3060	2970.3	89.6	7051	8298.9	1247.9	11890	10731.7	1158.2	2496 (11%)
X	1942	1942.9	0.9	5309	5428.3	119.3	7140	7019.7	120.2	240 (2%)
Total	1587 (9.9%)			15752 (35%)			10110 (17.48%)			27449 (23%)
G	2922	3120.5	198.5	9783	10459.0	676.0	4912	4037.4	874.5	1749 (10%)
R	1221	1457.2	236.2	4135	4884.2	749.2	2871	1885.4	985.5	1971 (23%)
O	1463	1479.7	16.7	4607	4959.6	352.6	2284	1914.5	369.4	739 (9%)
E	1849	1937.8	88.8	5734	6494.9	760.9	3357	2507.2	849.7	1699 (15%)
I	2693	2740.7	47.7	8099	9186.1	1087.1	4681	3546.0	1134.9	2269 (14%)
S	2337	2628.2	291.2	7941	8809.1	868.1	4560	3400.5	1159.4	2319 (16%)
Q	3459	3409.0	49.9	10712	11426.1	714.1	5075	4410.7	664.2	1428 (7%)
P	4063	4048.8	14.1	14095	13570.5	524.4	4700	5238.5	538.5	1077 (5%)
H	2999	2728.7	270.2	9961	9145.8	815.1	2445	3530.4	1085.4	2171 (14%)
Y	2192	1921.6	270.3	7344	6440.9	903.0	1313	2486.3	1173.3	2346 (22%)
D	1813	2063.9	250.9	8634	6917.6	1716.3	1205	2670.3	1465.3	3432 (29%)
U	2274	2023.9	250.0	7853	6783.5	1069.4	1299	2618.5	1319.5	2639 (23%)
F	2216	1940.4	275.5	6684	6503.8	180.1	2055	2510.6	455.6	911 (8%)
Total	2260 (7.2%)			10417 (9.6%)			12075 (29%)			24752 (14%)
J	1883	1845.7	37.2	5804	6090.7	286.7	2468	2218.5	249.4	573 (6%)
K	3795	3763.9	31.0	13069	12420.8	648.1	3845	4524.1	679.1	1358 (6%)
C	1815	1779.7	35.2	5563	5873.0	310.0	2414	2139.2	274.7	620 (6%)
B	5151	5114.5	36.4	17764	16877.8	886.1	5225	6147.5	922.5	1845 (6%)
Z	4393	4532.9	139.9	14021	14958.5	937.5	6526	5448.5	1077.4	2155 (9%)
Total	280 (1.6%)			3068 (5.4%)			3204 (15.6%)			6552 (7%)
